# Role of Inflammatory Cytokines Interleukin-1β and Interleukin-6 in Carcinogenesis, with Particular Emphasis on Gastroenteropancreatic Neuroendocrine Neoplasms

**DOI:** 10.3390/cancers18142257

**Published:** 2026-07-14

**Authors:** Izabella Ryguła, Violetta Rosiek, Beata Kos-Kudła

**Affiliations:** Department of Endocrinology and Neuroendocrine Tumors, Department of Pathophysiology and Endocrinology, Medical University of Silesia, 40-514 Katowice, Poland

**Keywords:** interleukin-1 beta, interleukin-6, inflammation, carcinogenesis, tumour microenvironment, neuroendocrine neoplasms, gastroenteropancreatic tumours, cytokine signaling

## Abstract

Inflammation is a hallmark of cancer and contributes to tumour initiation, progression, and therapeutic resistance. Among inflammatory mediators, interleukin-1β (IL-1β) and interleukin-6 (IL-6) are central cytokines linking innate immune responses with oncogenic signalling networks. This narrative review summarizes current experimental and clinical evidence regarding the role of IL-1β and IL-6 in carcinogenesis, with particular emphasis on gastroenteropancreatic neuroendocrine neoplasms (GEP-NENs). We discuss the molecular pathways associated with these cytokines, their interactions within the tumour microenvironment, and their contribution to tumour proliferation, angiogenesis, immune modulation, metastatic dissemination, and resistance to anticancer therapies. Particular attention is given to GEP-NENs, in which chronic inflammation and cytokine dysregulation may influence tumour behaviour, systemic inflammatory activity, and clinical outcomes. Accumulating evidence suggests that IL-6 may represent a promising exploratory biomarker associated with tumour burden, histological grade, disease progression, and systemic inflammation, whereas IL-1β appears to be more closely linked to local inflammatory signalling, microenvironmental remodelling, and potential susceptibility mechanisms. However, current evidence in GEP-NENs remains limited by small study cohorts, heterogeneous patient populations, variable analytical methodologies, and the lack of prospective validation. Further translational and clinical investigations are warranted to determine whether cytokine-based biomarkers and therapeutic modulation of inflammatory pathways may expand current diagnostic, prognostic, and therapeutic approaches in GEP-NENs.

## Simple Summary

Chronic inflammation is increasingly recognized as a key driver of cancer development and progression. Among inflammatory mediators, interleukin-1β (IL-1β) and interleukin-6 (IL-6) link immune dysregulation with tumour growth, invasion, and therapeutic resistance. Beyond their direct effects on cancer cells, these cytokines actively shape the tumour microenvironment by modulating immune responses, angiogenesis, and tissue remodelling. Gastroenteropancreatic neuroendocrine neoplasms (GEP-NENs) are biologically heterogeneous tumours in which the contribution of inflammatory signalling remains incompletely understood despite their increasing clinical relevance. This review summarizes current evidence on the role of IL-1β- and IL-6-driven pathways in carcinogenesis, with particular emphasis on GEP-NENs. Improved understanding of cytokine-mediated inflammatory mechanisms may facilitate the identification of clinically relevant biomarkers and support the development of future translational and therapeutic strategies.

## 1. Introduction

Advances in cancer biology over recent decades have established a close and bidirectional relationship between the immune system and both normal and malignant tissues [1]. Chronic inflammation is now recognized as a critical enabling factor in carcinogenesis, mediated through a complex network of pro-inflammatory cytokines and signalling pathways, among which interleukin-1β (IL-1β) and interleukin-6 (IL-6) play central roles [2,3]. Sustained activation of inflammatory signalling promotes multiple processes associated with malignant transformation, including enhanced proliferation, angiogenesis, immune evasion, and resistance to anticancer therapy. In parallel, tumour cells actively remodel the inflammatory tumour microenvironment (TME) through the secretion of cytokines and chemokines that perpetuate and amplify these tumour-promoting processes [4,5].

IL-1β functions as a major upstream regulator of inflammatory responses and exerts its biological effects primarily through activation of the nuclear factor-κB (NF-κB) signalling pathway, leading to transcription of genes involved in cell survival, invasion, migration, and amplification of inflammatory signalling [6,7]. By contrast, IL-6 signals through its receptor complex composed of interleukin-6 receptor (IL-6R) and the signal-transducing subunit glycoprotein 130 (gp130), predominantly activating the Janus kinase/signal transducer and activator of transcription 3 (JAK/STAT3) pathway. Persistent activation of this cascade promotes tumour cell proliferation, angiogenesis, metastatic dissemination, and resistance to anticancer therapies across multiple malignancies [8,9]. Importantly, NF-κB and STAT3 signalling are functionally interconnected and participate in positive feedback loops that sustain chronic inflammation within the TME [10].

Neuroendocrine neoplasms (NENs) arise from the diffuse neuroendocrine cell system and may develop in multiple organs. The gastrointestinal tract and pancreas constitute the most common primary sites, with gastroenteropancreatic neuroendocrine neoplasms (GEP-NENs) accounting for more than two-thirds of all NEN cases. Over recent decades, the incidence of NENs has steadily increased and is currently estimated at 8.52 cases per 100,000 individuals annually for all NENs and 6.1 cases per 100,000 individuals annually for GEP-NENs [11,12]. These neoplasms exhibit substantial biological heterogeneity, ranging from well-differentiated, indolent neuroendocrine tumours (NETs) to highly aggressive, poorly differentiated neuroendocrine carcinomas (NECs), resulting in diverse clinical behaviour and considerable therapeutic challenges.

This narrative review aims to summarize and critically evaluate current evidence regarding the role of IL-1β and IL-6 in carcinogenesis, with particular emphasis on their involvement in the development and progression of GEP-NENs. The combined focus on IL-1β and IL-6 in GEP-NENs is justified by the fact that both cytokines are key mediators of inflammation-driven carcinogenesis and may connect chronic inflammatory signalling with immune regulation, stromal activation, angiogenesis, tumour progression, and biomarker development. This focus is particularly relevant because GEP-NENs are biologically heterogeneous neoplasms, and the limited available evidence suggests that cytokine-related profiles may differ according to pancreatic versus gastrointestinal origin, functional versus non-functional status, and well-differentiated NET versus poorly differentiated NEC morphology. Given the limited and heterogeneous nature of GEP-NEN-specific evidence, mechanistic findings from colorectal cancer, pancreatic ductal adenocarcinoma, and other solid tumours are discussed only as supportive biological context and are not treated as established evidence in GEP-NENs.

Recent inflammatory models further support the concept that IL-1β and IL-6 should be interpreted within broader inflammatory networks rather than as isolated biomarkers. Multiplex serum–tissue profiling and fibroblast-stimulation studies have shown coordinated cytokine and chemokine responses, including IL-6 and IL-1β, highlighting the importance of stromal-cell activation, systemic–local biomarker discordance, and microenvironment-dependent inflammatory signalling [13,14]. Although these findings cannot be directly extrapolated to GEP-NENs, they provide biological rationale for evaluating IL-1β and IL-6 as components of tumour–stroma communication and for identifying evidence gaps in neuroendocrine tumour-specific studies.

A literature search was conducted using the PubMed, MEDLINE, and Google Scholar databases to identify studies investigating the role of IL-1β and IL-6 in cancer biology, with particular emphasis on neuroendocrine neoplasms. The search strategy included combinations of the following keywords: “inflammation”, “interleukin-1β”, “interleukin-6”, “carcinogenesis”, “neuroendocrine neoplasm”, “gastroenteropancreatic neuroendocrine neoplasms”, “GEP-NENs” and “tumour microenvironment”. Eligible publications included original research articles and review papers in which inflammatory processes and/or IL-1β and IL-6 constituted a primary focus. Editorials, commentaries, methodological reports, and letters to the editor were excluded. Study selection was performed in three sequential stages: title and abstract screening, full-text evaluation, and qualitative synthesis of extracted data. Given the heterogeneity of the available evidence, including experimental studies, retrospective clinical cohorts, immunohistochemical analyses, and translational investigations, a narrative rather than systematic review approach was adopted.

## 2. Molecular Mechanisms of Interleukin-1β and Interleukin-6

IL-1β and IL-6 are central mediators of inflammatory responses and play pivotal roles in coordinating immune, stromal, and epithelial cell functions. Although these cytokines differ substantially in their biosynthesis, secretion, and signalling mechanisms, they frequently cooperate within tissues affected by chronic inflammation, including the tumour microenvironment (TME) [15,16].

### 2.1. IL-1β

IL-1β is synthesized as an inactive precursor, pro-IL-1β, predominantly by myeloid-derived cells of the innate immune system, including monocytes, macrophages, and neutrophils [17]. Transcription of the IL1B gene is primarily induced through the activation of pattern-recognition receptors (PRRs), including Toll-like receptors (TLRs), following recognition of pathogen-associated molecular patterns (PAMPs), damage-associated molecular patterns (DAMPs), or stimulation by other pro-inflammatory cytokines [18,19]. These signals converge on intracellular pathways leading to the activation of NF-κB and the subsequent upregulation of pro-inflammatory genes, including IL1B itself [20].

The release of biologically active IL-1β requires a second activation signal promoting caspase-1 activation. Caspase-1 subsequently cleaves inactive pro-IL-1β (31 kDa) into its mature and biologically active form (17 kDa). This process occurs within a multiprotein complex termed the inflammasome, most commonly the NOD-like receptor family pyrin domain-containing 3 (NLRP3) inflammasome [21,22,23].

Unlike many other cytokines, IL-1β is secreted through non-classical pathways independent of the endoplasmic reticulum and Golgi apparatus [17,24]. Several mechanisms of IL-1β release have been proposed, including vesicular secretion via autophagolysosomes, microvesicles, and exosomes, as well as release through pores formed by gasdermin D (GSDMD) [18,19,23]. Formation of GSDMD pores within the plasma membrane triggers pyroptosis, a pro-inflammatory form of programmed cell death resulting in extracellular release of cytoplasmic contents, including IL-1β [25,26,27]. Importantly, GSDMD itself is activated by caspase-1, thereby functionally linking inflammasome activation to both cytokine secretion and inflammatory cell death [28].

Following release, IL-1β exerts its biological effects through receptors belonging to the Toll-like receptor/interleukin-1 receptor (TLR/IL-1R) superfamily, characterized by the presence of a cytoplasmic Toll/interleukin-1 receptor (TIR) domain. Two principal receptors have been identified: interleukin-1 receptor type 1 (IL-1R1) and interleukin-1 receptor type 2 (IL-1R2). Both receptors contain three extracellular immunoglobulin-like domains and interact with interleukin-1 receptor accessory protein (IL-1RAcP). However, only the IL-1β/IL-1R1/IL-1RAcP complex initiates intracellular signalling. In contrast, IL-1R2 lacks the intracellular TIR domain and therefore functions primarily as a decoy receptor by sequestering IL-1β and IL-1RAcP, thereby limiting downstream signal propagation [29]. In addition, the naturally occurring interleukin-1 receptor antagonist (IL-1Ra) binds IL-1R1 with high affinity without recruiting IL-1RAcP, effectively suppressing IL-1 signalling [6,29,30].

Engagement of IL-1R1 and IL-1RAcP results in recruitment of myeloid differentiation primary response 88 (MyD88) through TIR-domain interactions, followed by the assembly of a signalling complex containing interleukin-1 receptor-associated kinase 4 (IRAK4), IRAK1, IRAK2, and tumour necrosis factor receptor-associated factor 6 (TRAF6). TRAF6 subsequently activates transforming growth factor-beta-activated kinase 1 (TAK1), which stimulates two major downstream pathways: mitogen-activated protein kinases (MAPKs), including p38 and c-Jun N-terminal kinase (JNK), and NF-κB. Phosphorylation and degradation of the NF-κB inhibitor kappa B (IκB) permit translocation of the NF-κB p50/p65 complex into the nucleus, where it induces transcription of genes involved in inflammation, cell survival, invasion, and tissue remodelling [31].

Overall, activation of IL-1R1 signalling induces expression of cytokines, chemokines, and growth factors regulating proliferation, survival, and functional properties of immune, stromal, and tumour cells. The biological consequences of IL-1β signalling are highly context-dependent and influenced by tissue-specific receptor expression patterns and the balance between IL-1β and its endogenous antagonists [23].

### 2.2. IL-6

IL-6 is a pleiotropic glycoprotein produced by a broad spectrum of cell types, including monocytes, T lymphocytes and B lymphocytes, keratinocytes, fibroblasts, adipocytes, stromal cells, epithelial cells, muscle cells, and multiple tumour cell populations [32,33]. Expression of the IL6 gene may be induced by numerous stimuli, including TLR ligands, pro-inflammatory cytokines such as IL-1 and TNF-α, reactive oxygen species (ROS), and Zn^2+^ ions. In addition, IL-6 expression is subject to epigenetic regulation [34,35,36,37,38,39].

Three principal modes of IL-6 signalling have been described: classic signalling, trans-signalling, and trans-presentation [16,40]. In classic signalling, IL-6 binds to membrane-bound IL-6R (mIL-6R), forming a complex that associates with gp130, resulting in receptor dimerization and initiation of intracellular signalling. During trans-signalling, IL-6 interacts with soluble IL-6R (sIL-6R) generated through proteolytic cleavage or alternative splicing. The resulting IL-6/sIL-6R complex can subsequently activate gp130 on cells lacking mIL-6R expression, thereby substantially broadening the spectrum of IL-6-responsive cell types [41,42]. The third mechanism, termed trans-presentation or cluster signalling, occurs when IL-6 bound to mIL-6R on one cell activates gp130 on a neighbouring target cell [43].

Following receptor engagement, the IL-6/IL-6R/gp130 complex activates janus kinase (JAK) kinases, which phosphorylate tyrosine residues on gp130 and create docking sites for signal transducer and activator of transcription (STAT) proteins. STAT3 subsequently undergoes phosphorylation, dimerization, and nuclear translocation, where it regulates transcription of genes involved in tumour cell proliferation, survival, metastasis, angiogenesis, and inflammation [44,45,46]. Among the major STAT3 target genes is suppressor of cytokine signalling 3 (SOCS3), which functions as a negative feedback regulator limiting excessive JAK/STAT activation. Additional regulation is mediated by protein inhibitors of activated STAT (PIAS), which inhibit activated STAT dimers through interference with DNA binding [47,48].

Beyond the JAK/STAT3 axis, IL-6 signalling also activates additional intracellular pathways, including MAPK and phosphoinositide 3-kinase/protein kinase B (PI3K/Akt) signalling. MAPK activation contributes to regulation of gene expression associated with cell proliferation, acute-phase protein synthesis, and cellular stress responses. The activation of PI3K leads to generation of phosphatidylinositol-3,4,5-trisphosphate (PIP3) and subsequent Akt activation, promoting cell survival, growth, and metabolic adaptation. Collectively, these pathways underlie the pleiotropic effects of IL-6 in both physiological inflammatory responses and cancer development [32,49].

Taken together, IL-1β functions primarily as an early inflammatory trigger inducing production of secondary mediators, including IL-6, whereas IL-6—particularly through trans-signalling—sustains and amplifies inflammatory responses across a broader spectrum of cell types extending beyond classical immune populations. At the intracellular level, this coordinated activity involves activation of the IL-1β → MyD88 → IRAK → TRAF6 → TAK1 → NF-κB/MAPK axis and the IL-6 → gp130 → JAK → STAT3/MAPK/PI3K axis. Persistent cooperation between these pathways promotes expression of pro-inflammatory and pro-tumourigenic genes and, in the setting of chronic inflammation, contributes to remodelling of the TME and malignant transformation [1,40,50,51,52].

## 3. Role of IL-1β and IL-6 in Carcinogenesis

### 3.1. IL-1β and Carcinogenesis

IL-1β plays a fundamental role in carcinogenesis. Its pleiotropic effects contribute to the maintenance of chronic inflammation, promote tumour invasion and metastatic dissemination, and facilitate the development of resistance to anticancer therapies [23,53,54].

#### 3.1.1. Inflammatory Context and Tumour Initiation

IL-1β is a major pro-inflammatory cytokine and a central mediator of immune and inflammatory responses. The binding of IL-1β to IL-1R1 activates intracellular signalling cascades culminating in the activation of NF-κB and MAPKs, including JNK, p38, and extracellular signal-regulated kinase (ERK) [55]. Carcinogenesis and metastatic dissemination have been compared to processes of persistent tissue injury and dysregulated wound healing, both sustained by chronic inflammatory signalling involving members of the IL-1 cytokine family [54,55].

Experimental murine studies have demonstrated that IL-1β overexpression in gastric epithelial cells induces chronic gastritis and promotes formation of premalignant lesions in the stomach and pancreas [7,56]. Additional preclinical evidence indicates that IL-1β-driven inflammation enhances both the development and invasiveness of chemically induced tumours, whereas fibrosarcoma progression is delayed in IL-1β-deficient mice, further underscoring the contribution of this cytokine to tumour initiation and progression [57].

**Figure 1:**
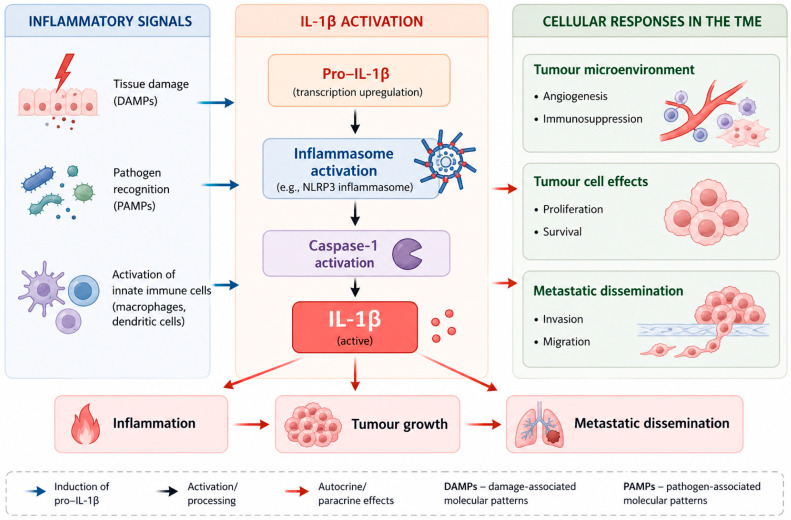
IL-1β signalling pathways in carcinogenesis. Schematic overview of IL-1β activation and its role in tumour-promoting inflammation. Inflammatory stimuli, including tissue damage, pathogen-associated molecular patterns (PAMPs), and activation of innate immune cells, induce transcription of pro-IL-1β. Subsequent inflammasome activation, particularly involving the NOD-like receptor family pyrin domain-containing (NLRP3) inflammasome, promotes caspase-1-dependent cleavage of pro-IL-1β into its biologically active form. Active IL-1β is released into the tumour microenvironment (TME), where it acts through autocrine and paracrine signalling on tumour, stromal, and immune cells. Activation of nuclear factor kappa B (NF-κB)—and mitogen-activated protein kinase (MAPK)-dependent pathways promotes angiogenesis, immune suppression, tumour cell proliferation and survival, invasion, and metastatic dissemination, thereby linking chronic inflammation with tumour progression.

The upstream role of IL-1β in linking inflammasome activation with NF-κB-dependent tumour-promoting programmes is schematically illustrated in Figure 1.

#### 3.1.2. Sources of IL-1β in the TME

IL-1β is produced and secreted by multiple cell populations within the TME. Major cellular sources include myeloid cells, particularly monocytes, macrophages, and neutrophils, although IL-1 family signalling may also involve stromal elements and, in certain contexts, tumour cells themselves [58,59,60,61,62].

Although immune cells are the predominant source of IL-1β within the tumour microenvironment, tumour-cell-derived IL-1β synthesis has also been documented in several malignancies, including melanoma, lung, breast, liver, colorectal, and prostate cancers [54,63,64,65,66]. In leukemic cells, autocrine IL-1β production has been associated with oncogenic Kristen rat sarcoma viral oncogene homologue (KRAS) mutations, highlighting a direct link between oncogenic signalling and inflammatory cytokine production [67].

Stromal cells: IL-1β produced by tumour-associated macrophages (TAMs) promotes tumour progression and angiogenesis in breast and lung cancers [68,69]. In pancreatic ductal adenocarcinoma (PDAC), IL-1β secreted by cancer-associated fibroblasts (CAFs) acts in an autocrine manner to activate IRAK4 and NF-κB signalling, thereby promoting fibrosis, tumour cell proliferation, survival, and resistance to chemotherapy [59]. Furthermore, IL-1β induced in pancreatic tumour cells following Toll-like receptor 4 (TLR4) activation stimulates pancreatic stellate cells, leading to the accumulation of M2-like TAMs within the TME and subsequent resistance to programmed cell death protein 1 (PD-1)-targeted immune checkpoint blockade [60]. Notably, TAMs themselves represent a major source of IL-1β in multiple tumour types [70].

#### 3.1.3. Mechanisms Promoting Tumour Progression

Angiogenesis and invasiveness: IL-1β plays a critical role in tumour invasiveness and angiogenesis by promoting neovascularization and tumour growth through the induction of pro-angiogenic mediators, including vascular endothelial growth factor (VEGF) [2,71]. IL-1β and VEGF may act synergistically, as their transcriptional programmes share common transcription factor binding sites [72]. In addition, IL-1β enhances invasive and metastatic potential through the activation of NF-κB and MAPK signalling, resulting in increased expression of matrix metalloproteinases, including matrix metalloproteinase-2 (MMP-2) and matrix metalloproteinase-9 (MMP-9) [73,74].

Formation of the metastatic niche: IL-1β induces the expression of endothelial adhesion molecules, including vascular cell adhesion molecule 1 (VCAM-1) and E-selectin, thereby facilitating adhesion and transmigration of circulating tumour cells into metastatic niches [75]. Tumour-derived IL-1β has also been shown to promote growth and colonization of breast cancer metastases within bone tissue, further emphasizing its role in establishing permissive metastatic microenvironments [76,77].

Immunosuppression: IL-1β actively suppresses antitumour immune responses by promoting the recruitment and activation of myeloid-derived suppressor cells (MDSCs) within the TME. These cells inhibit cytotoxic T-lymphocyte (CTLs) activity and contribute to immune evasion [78]. Importantly, MDSCs themselves may produce IL-1β, thereby creating a feed-forward inflammatory loop that further enhances tumour growth and metastatic dissemination [79].

Promotion of cancer stem cells: IL-1β has also been implicated in the maintenance and expansion of cancer stem cell (CSC) populations. IL-1β released by tumour cells stimulates mesenchymal stem cells to secrete prostaglandin E2 (PGE2) and cytokines such as IL-6 and interleukin-8 (IL-8), which subsequently activate β-catenin signalling in cancer cells and promote acquisition of CSC-associated properties [80]. Given the central role of CSCs in tumour initiation, progression, metastasis, and therapeutic resistance, these findings further support the contribution of IL-1β signalling to aggressive tumour behaviour [81].

#### 3.1.4. IL-1β and Resistance to Anticancer Therapy

Elevated IL-1β signalling has been associated with reduced efficacy of conventional anticancer therapies [82]. Cytotoxic agents such as doxorubicin and cisplatin may activate inflammasomes, particularly NLRP3, leading to the caspase-1-dependent pyroptotic death of mesothelial cells and subsequent release of IL-1β by tumour cells or MDSCs within the TME [83]. In pancreatic cancer, autocrine IL-1β production sustains constitutive NF-κB activation, thereby promoting resistance to chemotherapeutic agents including etoposide and doxorubicin [84,85]. Similarly, in patients with advanced non-small-cell lung cancer (NSCLC), elevated circulating IL-1β levels have been associated with shorter overall survival and progression-free survival following platinum-based chemotherapy [86].

Selected original studies investigating the role of IL-1β in carcinogenesis are summarized in Table 1.

### 3.2. IL-6 and Carcinogenesis

**Figure 2:**
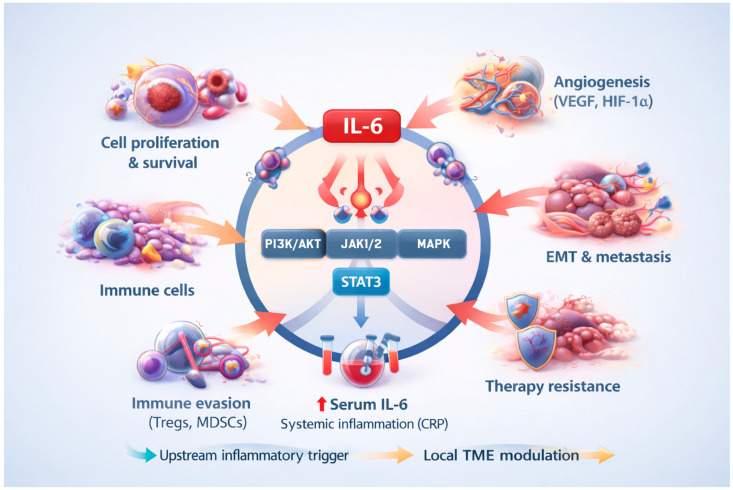
IL-6-mediated signalling pathways in cancer progression and systemic inflammation. Schematic representation of IL-6-mediated signalling and its role in tumour progression and systemic inflammatory responses. IL-6 binds to its receptor complex consisting of membrane-bound or soluble IL-6 receptor (IL-6R) and the signal-transducing subunit glycoprotein 130 (gp130), leading primarily to activation of the Janus kinase/signal transducer and activator of transcription 3 (JAK/STAT3) pathway, with additional involvement of mitogen-activated protein kinase (MAPK) and phosphoinositide 3-kinase/protein kinase B (PI3K/Akt) signalling. Sustained STAT3 activation promotes transcription of genes associated with tumour cell proliferation and survival, epithelial–mesenchymal transition (EMT), angiogenesis, and resistance to anticancer therapies. Within the tumour microenvironment (TME), IL-6 modulates immune cell function by promoting expansion of immunosuppressive populations, including regulatory T cells (Tregs) and myeloid-derived suppressor cells (MDSCs), thereby facilitating immune evasion. Elevated circulating IL-6 additionally contributes to systemic inflammation and increased C-reactive protein (CRP) levels. Collectively, IL-6 integrates local tumour-promoting signalling with systemic inflammatory responses, linking chronic inflammation with tumour progression and therapeutic resistance. Arrows indicate activation, stimulation, or the direction of IL-6-mediated signalling effects.

IL-6 is a pleiotropic pro-inflammatory cytokine widely recognized as a key molecular link between chronic inflammation and cancer development. The diverse biological effects of IL-6 signalling connecting chronic inflammation with tumour progression, immune evasion, angiogenesis, and systemic inflammatory responses are summarized in Figure 2.

Excessive IL-6 production within the TME contributes to tumour initiation, progression, and metastatic dissemination across a broad spectrum of malignancies [40]. Elevated circulating IL-6 levels in patients with breast, colorectal, hepatocellular, and soft tissue sarcomas have consistently been associated with unfavourable prognosis and reduced overall survival [97,98,99,100].

#### 3.2.1. Pro-Carcinogenic Activity and Tumour Progression

IL-6 influences virtually all stages of tumour development by regulating tumour cell proliferation, survival, angiogenesis, invasion, and metastatic spread [32,34,101].

Proliferation and survival: The principal oncogenic mechanism of IL-6 involves activation of the JAK/STAT3 signalling axis. Activated STAT3 induces transcription of anti-apoptotic genes, including B-cell lymphoma 2 (BCL2), B-cell lymphoma-extra large (BCL-XL), myeloid cell leukemia 1 (MCL1), and survivin, as well as genes regulating cell-cycle progression, such as c-MYC and cyclins [34,102,103,104,105,106,107,108]. STAT3 activation appears essential for survival of intestinal epithelial cells during inflammation-associated carcinogenesis [109]. In parallel, IL-6 activates PI3K/Akt and ERK/MAPK signalling, further promoting tumour cell survival and proliferation [110,111,112].

Angiogenesis and metastasis: IL-6 is also a potent driver of tumour invasion and metastatic dissemination. Through STAT3-dependent mechanisms, IL-6 upregulates VEGF and hypoxia-inducible factor 1-alpha (HIF-1α), thereby promoting angiogenesis [113,114,115,116]. In addition, IL-6 contributes to EMT, a critical process underlying metastatic progression. In gastric cancer, activation of the IL-6/JAK2/STAT3 axis facilitates EMT and tumour invasion [117], whereas in colorectal cancer IL-6/STAT3 signalling induces the upregulation of leucine-rich alpha-2-glycoprotein 1 (LRG1), thereby enhancing metastatic potential [118]. In lung cancer, CAF-derived IL-6 released in a Musashi-2-dependent manner promotes EMT through paracrine signalling [119]. IL-6 further contributes to bone metastasis by promoting osteolysis and facilitating homing of tumour cells to the bone marrow niche [120,121].

#### 3.2.2. Remodelling of the TME

IL-6 is a major regulator of tumour-promoting inflammation and plays a central role in shaping both innate and adaptive immune responses within the TME. IL-6 present in the TME is produced by multiple cell populations, including tumour cells, CAFs, TAMs, MDSCs, dendritic cells (DCs), and adipocytes [101,122,123,124]. Through autocrine and paracrine signalling, IL-6 orchestrates cellular interactions that favour tumour growth, immune evasion, and therapeutic resistance.

Cancer-associated fibroblasts. CAFs frequently represent a dominant source of IL-6 within the TME. Elevated IL-6 production by CAFs has consistently been associated with unfavourable clinical outcomes across multiple tumour types [125,126]. CAF-derived IL-6 activates STAT3 and Akt signalling in tumour cells, thereby contributing to resistance to cytotoxic agents such as doxorubicin [127]. In addition, CAFs may release sIL-6R, which together with IL-6 induces TAMs to upregulate transformin growth factor-beta 1 (TGF-β1). This mechanism suppresses natural killer (NK) cell activity and promotes immune escape through impairment of antitumour immune surveillance [128].

Myeloid cells and regulatory T cells. IL-6 promotes the recruitment, expansion, and immunosuppressive polarization of MDSCs within the TME, frequently through STAT3-dependent upregulation of C-C chemokine receptor type 5 (CCR5) and arginase-1, resulting in suppression of effector T-cell responses. MDSCs further amplify tumour-promoting inflammation through IL-6 secretion, disrupting Th1 differentiation and reinforcing immunosuppressive signalling [129,130,131]. Moreover, IL-6, in cooperation with leukemia inhibitory factor (LIF), directs monocyte differentiation toward M2-like TAMs characterized by pronounced pro-tumourigenic and immunosuppressive properties [132,133]. TAM-derived IL-6 subsequently sustains STAT3 activation in tumour cells, thereby enhancing self-renewal capacity and promoting cancer stemness [134].

T lymphocytes and NK cells. Through activation of STAT3 and etinoic acid receptor-related orphan receptor gamma t (RORγt), IL-6 acts as a critical driver of differentiation of naïve cluster of differentiation 4-positive (CD4^+^) T cells into pro-inflammatory T helper 17 (Th17) cells. IL-6 may inhibit Treg generation through the suppression of forkhead box P3 (FoxP3) expression; however, in certain tumour contexts it can also enhance Treg expansion and functional activity, for example via PI3K/Akt signalling [49,135]. Tregs contribute to immune suppression through release of IL-10 and TGF-β1, thereby impairing cytotoxic T lymphocyte (CTL) activity and facilitating tumour immune evasion. In DCs, IL-6/STAT3 signalling induces expression of indoleamine 2,3-dioxygenase (IDO), further promoting Treg expansion and immune tolerance [136]. In addition, IL-6 and IL-8 released by tumour cells activate STAT3 signalling in NK cells, leading to downregulation of activating receptors such as natural cytotoxicity triggering receptor 3 (NKp30) and natural killer group 2D (NKG2D) and consequently reducing NK cell-mediated cytotoxicity [137].

#### 3.2.3. IL-6, Therapeutic Resistance, and Immune Evasion

IL-6 is a critical mediator of tumour-immune evasion and has been strongly associated with resistance to chemotherapy, radiotherapy, and immunotherapy [8,135]. Sustained IL-6 signalling reinforces pro-survival programmes, supports therapy-tolerant cellular states, and may contribute to malignant transformation through self-amplifying inflammatory circuits [138].

IL-6 weakens chemotherapy-induced antitumour immunity [126,139]. The binding of IL-6 to its receptor activates JAK2, which may promote phosphorylation of beclin 1 (BECN1), thereby inducing pro-survival autophagy and accelerating resistance to chemotherapeutic agents [140]. In addition, CAF-derived IL-6 activates STAT3 signalling and contributes to radioresistance in breast cancer cells [141].

IL-6 also contributes to resistance to immunotherapy. It promotes T-cell exhaustion characterized by increased expression of inhibitory receptors, including PD-1, cytotoxic T-lymphocyte-associated protein 4 (CTLA-4), and lymphocyte activation gene 3 (LAG-3). Furthermore, IL-6 stimulates the JAK1/STAT3 pathway, increasing levels of glycosylated PD-L1, impairing T-cell function, and reducing immunotherapeutic efficacy [142]. Clinically, elevated circulating IL-6 levels have been proposed as predictive markers of poor response to immune checkpoint blockade (ICB), including anti-PD-1/PD-L1 and anti-CTLA-4 therapies [143,144,145,146]. Preclinical evidence further supports a functional link between IL-6 signalling and immune escape, as the combined targeting of IL-6-driven inflammation and the PD-1/PD-L1 axis has demonstrated greater antitumour efficacy than either strategy alone [147].

Selected original studies investigating the role of IL-6 in carcinogenesis are summarized in Table 2.

## 4. IL-1β and IL-6 in the Tumour Microenvironment

While Section 3 focused on the broader roles of IL-1β and IL-6 in carcinogenesis, this section specifically addresses cytokine-mediated intercellular communication within the tumour microenvironment.

The TME is a highly dynamic and adaptive ecosystem composed of malignant cells, stromal elements, immune infiltrates, endothelial cells, and extracellular matrix components. Rather than acting as isolated mediators, IL-1β and IL-6 function as central organizers of tumour–stroma communication and coordinate inflammatory signalling networks that shape the structural, metabolic, and immunological landscape of the TME [54,58,68,101]. As discussed in previous sections, the sustained activation of IL-1β- and IL-6-dependent pathways promotes chronic inflammation, angiogenesis, immune suppression, EMT, and resistance to anticancer therapies [59,69,119,134]. Accordingly, this section focuses on the integrative role of these cytokines in orchestrating multicellular interactions within the TME.

### 4.1. Paracrine and Autocrine Cytokine Loops in the TME

A defining feature of IL-1β- and IL-6-driven tumour inflammation is the establishment of self-sustaining paracrine and autocrine signalling loops. Tumour cells, CAFs, TAMs, and MDSCs engage in reciprocal cytokine exchange, thereby reinforcing a pro-inflammatory and tumour-supportive microenvironment [58,59,60,61,62,70,79]. IL-1β released by tumour cells or myeloid populations induces IL-6 production in stromal and immune cells, whereas IL-6 signalling may sustain IL-1β expression through STAT3- and NF-κB-dependent transcriptional programmes [40,54,101]. These feedback loops stabilize chronic inflammatory signalling and enable tumours to maintain growth-promoting conditions even after the initiating inflammatory stimulus has subsided.

Importantly, IL-1β and IL-6 do not act in isolation, but cooperate with other inflammatory mediators within broader cytokine networks. TNF-α may amplify NF-κB-dependent inflammatory signalling and promote secondary induction of IL-1β and IL-6, thereby reinforcing tumour-promoting inflammation. IL-8 can act downstream of IL-1β- and IL-6-related signalling to support myeloid-cell recruitment, angiogenesis, invasion, and therapy-resistant phenotypes [80,137]. In parallel, TGF-β may cooperate with IL-6- and IL-1β-driven inflammation by promoting stromal remodelling, extracellular matrix deposition, immune suppression, and EMT-like programmes [128]. In gastroenteropancreatic neuroendocrine neoplasms (GEP-NENs), direct mechanistic validation of these synergistic interactions remains limited; however, tissue-based studies showing prognostic relevance of TNF-α and high IL-6 expression, together with serum studies demonstrating altered IL-1β, IL-6, IL-8, and TNF levels in NET patients, support the concept that IL-1β and IL-6 should be interpreted as components of a broader inflammatory network rather than isolated biomarkers [158,159].

### 4.2. Network-Level Cytokine Interactions Within the Tumour Microenvironment

Within the TME, IL-1β and IL-6 shape reciprocal communication among tumour cells, cancer-associated fibroblasts (CAFs), tumour-associated macrophages (TAMs), myeloid-derived suppressor cells (MDSCs), endothelial cells, antigen-presenting cells, and lymphocyte populations. Rather than acting through isolated linear pathways, these cytokines participate in interconnected inflammatory circuits that coordinate stromal remodelling, myeloid-cell polarization, impaired cytotoxic lymphocyte activity, angiogenesis, vascular niche formation, and therapy-tolerant tumour states [97,101,102,103,104,105,106,110,111,112,113].

IL-1β may act as an upstream inflammatory trigger by activating NF-κB- and MAPK-dependent signalling in tumour, stromal, and immune cells. This can promote the secondary production of IL-6, IL-8, VEGF, matrix-remodelling enzymes, and other mediators involved in immune-cell recruitment, extracellular matrix remodelling, and tumour invasion [71,72,73,74,75,76,77,78,79,80]. IL-6, in turn, may sustain these inflammatory circuits through JAK/STAT3-dependent transcriptional programmes, supporting tumour-cell survival, angiogenesis, immune suppression, and resistance to anticancer therapies [101,122,123,124,125,126,127,128,129,130,131,132,133,134,135,136,137]. In this way, IL-1β- and IL-6-related signalling may reinforce a self-sustaining inflammatory network within the TME.

These cytokine-mediated interactions also influence vascular and metastatic niche formation. IL-1β and IL-6 can act on endothelial cells, CAFs, macrophages, and bone marrow-derived cells to promote neovascularization, endothelial activation, tumour-cell adhesion, and stromal changes that facilitate tumour-cell survival and colonization at distant sites [75,76,77,113,114,115,116,117,118,119,120,121]. Their relevance therefore extends beyond direct effects on tumour-cell proliferation and includes the organization of a supportive stromal, vascular, and immune microenvironment.

Finally, IL-1β and IL-6 may contribute to immune escape and treatment resistance by linking tumour cells with antigen-presenting cells, myeloid populations, T cells, NK cells, and stromal elements. The IL-1β-driven recruitment of immunosuppressive myeloid populations and IL-6/STAT3-mediated suppression of antitumour immune responses may reduce cytotoxic lymphocyte and NK-cell activity, promote T-cell dysfunction, and support resistance to chemotherapy, radiotherapy, and immune checkpoint blockade [78,79,128,129,130,131,132,133,134,135,136,137,138,139,140,141,142,143,144,145,146,147]. Although these network-level mechanisms have been extensively characterized in several solid malignancies, direct mechanistic evidence in GEP-NENs remains limited. Consequently, these concepts should currently be regarded as biologically plausible hypotheses for neuroendocrine neoplasms rather than definitively established GEP-NEN-specific mechanisms.

## 5. IL-1β and IL-6 in Gastroenteropancreatic Neuroendocrine Neoplasms

Neuroendocrine neoplasms (NENs) represent a heterogeneous group of tumours arising from the diffuse neuroendocrine cell system and may develop in multiple organs. Their incidence has steadily increased and currently reaches 8.52 cases per 100,000 individuals annually, with more than two-thirds originating in the gastroenteropancreatic tract [11,12]. Gastroenteropancreatic neuroendocrine neoplasms (GEP-NENs) comprise a biologically diverse group of tumours, including well-differentiated neuroendocrine tumours (NETs) and poorly differentiated neuroendocrine carcinomas (NECs). According to the World Health Organization (WHO) classification, these entities are stratified according to tumour differentiation, mitotic count, and proliferative activity assessed by the Ki-67 index [160]. This classification is clinically relevant, as GEP-NENs range from indolent and slowly progressive NET G1/G2 lesions to highly aggressive NET G3 and NECs.

In this context, IL-1β and IL-6 are of particular interest as mediators linking chronic inflammation, tumour-promoting signalling, and remodelling of the TME in GEP-NENs [161,162]. Their potential role in GEP-NEN biology may be considered at multiple levels, including systemic inflammatory activity, local tumour tissue expression, genetic susceptibility, tumour grade, clinical aggressiveness, and possible therapeutic relevance [158,159,163,164,165].

The reciprocal relationship between inflammatory cytokine signalling and neuroendocrine differentiation in GEP-NENs remains poorly defined. Classical neuroendocrine markers, such as chromogranin A and synaptophysin, are routinely used to confirm the neuroendocrine phenotype, but available studies have not established whether IL-1β or IL-6 directly regulate their expression. Current evidence instead suggests an association between inflammatory activation and neuroendocrine tumour biology: serum IL-1β and IL-6 were elevated in NET patients and IL-6 showed a weak positive correlation with chromogranin A [163]. Moreover, tissue-based studies of GEP-NENs confirmed neuroendocrine differentiation by chromogranin and synaptophysin expression while demonstrating local IL-1β- and IL-6-related inflammatory activity [159]. However, whether IL-1β or IL-6 directly modulate chromogranin A or synaptophysin expression, or whether these markers simply coexist with cytokine-driven inflammatory activity, remains unknown and requires mechanistic validation in GEP-NEN models.

### 5.1. Biological Rationale: Inflammatory Signalling in the GEP-NEN Microenvironment

Similarly to other solid tumours discussed in previous sections, GEP-NENs develop within a complex TME composed of neuroendocrine tumour cells, stromal cells, immune and inflammatory infiltrates, CAFs, blood and lymphatic vessels, extracellular matrix components, and soluble mediators such as cytokines, chemokines, and growth factors [161,166]. Chronic inflammatory signalling may contribute to tumour initiation, progression, angiogenesis, stromal remodelling, and immune escape [161,167,168,169].

IL-1β and IL-6 represent biologically plausible mediators in this setting because both cytokines connect inflammatory responses with oncogenic signalling pathways. IL-1β activates NF-κB-dependent signalling and may promote release of secondary inflammatory mediators, whereas IL-6 acts as a major activator of the JAK/STAT3 axis. In GEP-NENs, increased nuclear STAT3 expression and its association with metastatic status have been reported, suggesting that IL-6-related signalling may contribute to tumour progression [170]. Moreover, non-canonical Notch signalling has been implicated in NEN tumorigenesis through interactions with several oncogenic pathways, including NF-κB [171,172].

Clinical observations further support a relationship between systemic inflammation and disease burden in NENs. Papalou et al. reported a positive association between serum chromogranin A and C-reactive protein (CRP) concentrations in patients with NENs. Higher CRP levels were additionally associated with a significantly increased likelihood of metastatic disease compared with localized tumours [173]. Although CRP is not specific to IL-1β- or IL-6-related activity, these findings support the broader concept that systemic inflammatory activation may reflect tumour burden and disease activity in NENs.

Chronic inflammatory processes may also contribute to the excessive activation of enteroendocrine cells, resulting in hyperplasia and potentially creating conditions favourable for neoplastic transformation [162,174,175,176]. Within this context, IL-1β and IL-6 may be regarded as components of a broader inflammatory network linking tissue injury, immune activation, stromal remodelling, and neuroendocrine tumour biology.

However, it should be emphasized that much of the mechanistic framework linking IL-1β and IL-6 with tumour progression has been derived from studies performed in non-neuroendocrine malignancies. Although available clinical and translational observations support the relevance of these pathways in GEP-NENs, direct experimental evidence remains comparatively scarce.

### 5.2. Serum and Tissue Expression of IL-1β and IL-6: What Has Been Shown?

Available studies assessing IL-1β and IL-6 in GEP-NENs can broadly be divided into serum-based analyses and tissue-based studies. These approaches provide complementary, although not interchangeable, information. Serum cytokine concentrations may reflect systemic inflammatory activity and tumour burden, whereas tissue expression assessed by immunohistochemistry may more accurately reflect local inflammatory processes within the tumour and the surrounding TME.

Geisler et al. analyzed serum concentrations of multiple cytokines, including IL-1β and IL-6, in 43 patients with well-differentiated gastrointestinal neuroendocrine tumours. Both IL-1β and IL-6 levels were significantly elevated in patients compared with healthy controls, and IL-6 concentrations correlated significantly with tumour stage [158].

Budek et al. evaluated serum concentrations of chromogranin A and 48 cytokines, including IL-1β and IL-6, in 84 patients with NENs, of whom 54 had GEP-NENs. Serum concentrations of IL-1β and IL-6 were significantly higher in patients than in controls, and positive correlations were observed between cytokine concentrations and chromogranin A levels [163]. Similarly, Strzelczyk et al. assessed serum concentrations of cytokines belonging to the IL-6 family in 80 patients with NENs, including 64 with GEP-NENs, and demonstrated significantly elevated IL-6 levels compared with healthy controls [177].

Tissue-based observations further support the presence of IL-1β- and IL-6-associated inflammatory activity in GEP-NENs. Herman Mahečić et al. evaluated the immunohistochemical expression of four pro-inflammatory cytokines, including IL-1β and IL-6, in tumour tissue specimens obtained from 43 patients with GEP-NENs. Most tumours exhibited high expression of both IL-6 and IL-1β, while IL-6 expression increased with tumour grade [159].

The interpretation of these findings should be approached with caution. Most currently available studies evaluating IL-1β and IL-6 in GEP-NENs are retrospective, involve relatively small patient cohorts, and frequently include tumours originating from different anatomical sites. Moreover, the biological heterogeneity of GEP-NENs complicates direct comparison across studies and may contribute to inconsistencies in reported associations.

Pereira et al. examined expression of several inflammatory and metabolic markers, including IL-6, in tumour tissues from patients with well-differentiated GEP-NENs and metabolic syndrome. Higher IL-6 expression within GEP-NEN tissue positively correlated with disease progression. Furthermore, peritumoural IL-6 expression was higher in patients with GEP-NENs and low high-density lipoprotein (HDL) cholesterol concentrations [178].

Collectively, currently available serum- and tissue-based studies suggest that IL-6, and to a lesser extent IL-1β, may be elevated in patients with GEP-NENs and may reflect tumour burden, tumour grade, disease progression, or local inflammatory remodelling of the TME.

Nevertheless, these studies primarily demonstrate associations rather than causality. Whether IL-1β and IL-6 directly contribute to tumour progression or merely reflect tumour-associated inflammatory activity remains uncertain and requires further investigation in prospective and mechanistic studies.

### 5.3. Pancreatic Versus Gastrointestinal NENs and Functioning Versus Non-Functioning Tumours

An important clinical question is whether IL-1β- and IL-6-related findings differ between pancreatic and gastrointestinal NENs, as well as between functioning and non-functioning tumours. These subgroups differ substantially in clinical presentation, hormonal activity, tumour biology, prognosis, and therapeutic management.

The distinction between pancreatic and gastrointestinal NENs may be particularly relevant in the context of IL-6-related signalling. In a study evaluating IL-6 −174 polymorphisms in 80 patients with GEP-NENs and 162 age- and sex-matched healthy controls, serum IL-6 concentrations were additionally analyzed according to IL-6 −174 genotypes. Among patients with non-functioning pancreatic NENs, only IL-6 −174 CG and GG genotypes, both associated with higher IL-6 expression, were identified, and genotype distribution differed significantly from that observed in functioning tumours. Across the entire GEP-NEN cohort, elevated serum IL-6 concentrations correlated with the GG genotype. Furthermore, patients with non-functioning pancreatic NENs exhibited significantly higher serum IL-6 concentrations than patients with functioning pancreatic NENs and gastrointestinal NENs [165].

These observations suggest that IL-6-related genetic and inflammatory profiles may differ according to tumour localization and hormonal functionality. In particular, non-functioning pancreatic NENs may represent a subgroup characterized by more pronounced IL-6-related inflammatory activity.

IL-1β polymorphisms have likewise been investigated predominantly in pancreatic NENs. Cigrovski Berković et al. isolated genomic DNA from peripheral blood samples obtained from 60 patients with pancreatic NENs and 60 healthy controls. Their analyses demonstrated a statistically significant association between the IL-1β −511C/T polymorphism and coexistence of CT/CT genotypes at positions −511 and +3954 with increased susceptibility to functioning pancreatic NENs. Conversely, the combined presence of CT and CC genotypes at positions −511 and +3954 of the IL1B gene was associated with increased predisposition to non-functioning pancreatic NENs [164].

Similarly, Karakaxas et al. conducted a case–control study demonstrating that the IL-1β −511 C/T polymorphism was more frequent in patients with pancreatic NENs than in healthy individuals. The −511 CT and TT genotypes, associated with higher IL-1β expression, were overrepresented in the pancreatic NEN group, suggesting a potential contribution of IL-1β to pancreatic NEN pathogenesis [179].

An additional limitation is that many studies combine pancreatic and gastrointestinal tumours despite their distinct biological characteristics, molecular profiles, and clinical behaviour. Consequently, it remains uncertain whether observed cytokine patterns reflect shared neuroendocrine biology or site-specific disease mechanisms.

### 5.4. NET Versus NEC and Grade-Dependent Inflammatory Signalling

Well-differentiated neuroendocrine tumours (NETs) and poorly differentiated neuroendocrine carcinomas (NECs) represent biologically distinct entities despite sharing neuroendocrine differentiation markers. According to the current WHO classification, these neoplasms differ substantially with respect to proliferative activity, genomic alterations, clinical behaviour, and prognosis [160]. Consequently, inflammatory signalling pathways may also differ considerably across the spectrum of GEP-NENs.

Available evidence suggests that IL-6 is more consistently associated with tumour aggressiveness than IL-1β. In the immunohistochemical study by Herman Mahečić et al., IL-6 expression showed an increasing trend with tumour grade, whereas IL-1β expression demonstrated less consistent clinicopathological associations [159]. Similarly, serum-based studies reported higher IL-6 concentrations in patients with advanced disease and greater tumour burden, supporting a relationship between IL-6 signalling and tumour progression [158,163].

Several biological mechanisms may explain these observations. Higher-grade NETs and NECs are characterized by increased proliferative activity, enhanced metabolic demands, tumour hypoxia, genomic instability, and more extensive stromal remodelling. These processes promote the activation of NF-κB- and STAT3-dependent inflammatory pathways, which are closely linked to IL-1β and IL-6 production. In particular, persistent activation of the JAK/STAT3 pathway has been reported in GEP-NENs and has been associated with metastatic disease, suggesting a potential role for IL-6-mediated signalling in tumour progression and dissemination [170].

Differences between NETs and NECs may also be reflected in the composition of the tumour microenvironment. Well-differentiated NETs generally exhibit slower growth kinetics and a relatively less inflammatory microenvironment, whereas NECs are frequently associated with extensive necrosis, increased immune-cell infiltration, and stronger activation of inflammatory signalling networks [161,166,167,168,169]. Such features may contribute to enhanced cytokine production and the amplification of tumour-promoting inflammatory circuits.

Nevertheless, current evidence remains limited. Importantly, direct evidence comparing inflammatory signalling across NET and NEC subtypes remains limited. Most available studies involve relatively small cohorts, frequently pool NETs and NECs within the same analyses, and often lack independent validation datasets. As a result, the extent to which IL-1β- and IL-6-related pathways differ according to tumour differentiation and grade remains incompletely defined [158,159,163]. Future studies incorporating larger, molecularly characterized cohorts and stratified analyses according to WHO grade and tumour differentiation will be essential to clarify the role of inflammatory cytokines across the biological spectrum of GEP-NENs.

The potential relationship between tumour differentiation, histological grade, and IL-1β/IL-6-related inflammatory signalling across the spectrum of GEP-NENs is summarized in Table 3.

### 5.5. Distinctive Features of the Neuroendocrine Tumour Microenvironment

The tumour microenvironment (TME) of GEP-NENs exhibits several distinctive features that differentiate these tumours from many conventional epithelial malignancies. Although the inflammatory landscape of GEP-NENs remains incompletely characterized, accumulating evidence suggests that tumour progression is not only influenced by intrinsic genetic alterations, but also by complex interactions among neuroendocrine tumour cells, stromal components, immune infiltrates, vascular structures, and soluble mediators, including inflammatory cytokines [161,166,167,168,169].

One of the hallmarks of well-differentiated GEP-NENs is their exceptionally rich vascular network. In contrast to many adenocarcinomas, where increasing aggressiveness is often accompanied by vascular destruction and hypoxia, low- and intermediate-grade NETs frequently demonstrate dense microvascularization and strong expression of angiogenic mediators. This phenomenon has been described as the “neuroendocrine paradox,” whereby highly vascularized tumours may nevertheless exhibit relatively indolent clinical behaviour [161,167]. Given the established role of IL-6 in angiogenic signalling through the induction of VEGF and related pathways, inflammatory cytokines may contribute to maintaining this highly vascular phenotype, although direct mechanistic evidence in GEP-NENs remains limited.

Another distinctive feature of the neuroendocrine microenvironment is its close association with hormone-producing cells and secretory activity. Unlike most non-neuroendocrine malignancies, functioning GEP-NENs may release biologically active hormones and peptides that influence both local and systemic inflammatory responses. Bioactive products, particularly serotonin, may influence local vascular, stromal, and fibrotic responses, whereas inflammatory cytokines may, in turn, modulate neuroendocrine-cell activation and secretory activity. However, direct evidence linking IL-1β or IL-6 to hormone secretion in functional GEP-NENs remains limited. In the study by Strzelczyk et al., serum IL-6 did not correlate with serotonin or 5-hydroxyindoleacetic acid (5-HIAA) levels, suggesting that circulating IL-6 may reflect systemic inflammatory or microenvironmental activity rather than serotonin-dependent hormonal activity alone [178]. Therefore, cytokine–hormone interactions in functioning GEP-NENs remain incompletely understood and require further clinically stratified validation.

The immune landscape of GEP-NENs also appears to differ according to tumour differentiation and grade. Well-differentiated NETs generally display relatively low tumour mutational burden, limited immune-cell infiltration, and a less inflammatory microenvironment, whereas poorly differentiated NECs frequently exhibit more pronounced immune activation, increased inflammatory infiltrates, and greater expression of immune-regulatory pathways [160,166,167,168,169]. These observations suggest that inflammatory cytokines may play distinct biological roles across the spectrum of neuroendocrine neoplasia.

Cancer-associated fibroblasts (CAFs), endothelial cells, tumour-associated macrophages, and other stromal components are increasingly recognized as important contributors to neuroendocrine tumour progression. Through the secretion of cytokines, chemokines, and extracellular matrix remodelling factors, these cells may create a tumour-supportive niche that promotes angiogenesis, tumour growth, and immune evasion [161,166,167,168,169]. While many mechanistic insights regarding CAF-derived IL-1β and IL-6 originate from studies in pancreatic adenocarcinoma and other solid tumours, similar stromal interactions may also occur in GEP-NENs and warrant further investigation.

Importantly, current knowledge regarding cytokine-mediated communication within the GEP-NEN microenvironment remains fragmentary [161,166,167,168,169]. Most available studies are retrospective, involve relatively small patient cohorts, and frequently combine tumours arising from different anatomical sites or pool biologically distinct NET and NEC populations [158,159,163,165,177,178]. Furthermore, independent validation cohorts are rarely available, and most data rely predominantly on immunohistochemical analyses or circulating biomarker measurements [158,159,163,177,178]. Consequently, many aspects of IL-1β- and IL-6-dependent signalling within neuroendocrine tumours remain biologically plausible but not yet directly demonstrated [161,166,167,168,169,170]. Future studies employing single-cell transcriptomics, spatial profiling technologies, multiplex tissue imaging, and larger prospective cohorts may provide a more comprehensive understanding of cytokine networks operating within the neuroendocrine tumour microenvironment. The main biological, clinical, and translational differences between IL-1β and IL-6 in GEP-NENs are summarized in Table 4.

Overall, the currently available evidence suggests that IL-6 has stronger support as a potential biomarker and therapeutic target in GEP-NENs than IL-1β. However, the limited number of studies directly evaluating IL-1β does not allow definitive conclusions regarding its clinical relevance.

**Figure 3:**
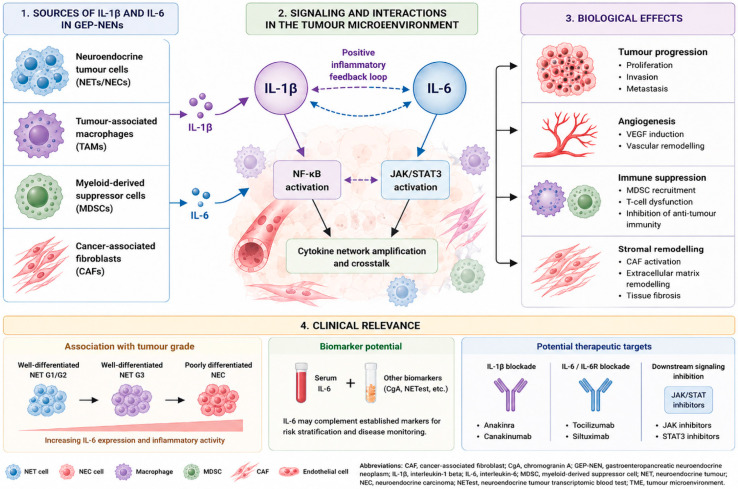
Proposed role of IL-1β and IL-6 within the gastroenteropancreatic neuroendocrine tumour microenvironment. Neuroendocrine tumour cells, tumour-associated macrophages, myeloid-derived suppressor cells, cancer-associated fibroblasts, endothelial cells, and other stromal components may contribute to IL-1β and IL-6 production. Through activation of NF-κB- and JAK/STAT3-dependent pathways, these cytokines promote tumour progression, angiogenesis, stromal remodelling, and immune suppression. Current evidence suggests that IL-6 is more consistently associated with tumour aggressiveness and higher tumour grade than IL-1β. Potential therapeutic strategies include blockade of IL-1β, IL-6/IL-6R, and downstream JAK/STAT signalling pathways. Solid arrows indicate activation, stimulation, or the direction of cytokine-mediated effects, whereas dashed arrows indicate feedback loops or crosstalk between signalling pathways.

The proposed interactions between IL-1β, IL-6, tumour cells, stromal components, and immune-cell populations within the GEP-NEN tumour microenvironment are summarized in Figure 3.

### 5.6. Emerging Molecular Profiling Approaches in GEP-NENs

Modern molecular profiling approaches have begun to refine understanding of tumour heterogeneity and tumour-immune interactions in neuroendocrine neoplasms. Most currently available evidence regarding IL-1β and IL-6 in GEP-NENs is derived from serum cytokine measurements, immunohistochemistry, or candidate-gene analyses. However, bulk transcriptomics, single-cell RNA sequencing, spatial transcriptomics, multiplex tissue imaging, and proteomic approaches now offer the possibility of identifying cytokine-associated pathways within specific tumour-, stromal-, endothelial-, and immune-cell compartments.

As a methodological and translational parallel, recent studies from non-neuroendocrine inflammatory models further illustrate why cytokine-focused research in GEP-NENs should move beyond isolated single-marker measurements. Multiplex serum–tissue profiling in experimental inflammatory models has demonstrated rapid and dynamic changes in multiple cytokines and chemokines, including IL-6, highlighting the importance of integrated assessment of systemic and local inflammatory responses [13]. Similarly, transcriptomic and protein-level analyses of tumour necrosis factor-alpha (TNF-α)-stimulated human fibroblasts have shown coordinated induction of broad pro-inflammatory cytokine and chemokine programmes, including IL-6 and IL-1β [14]. Although these studies were not performed in GEP-NENs, they provide mechanistic context for formulating translational hypotheses regarding cytokine-driven tumour–stroma communication, serum–tissue biomarker discordance, and the need for microenvironment-focused validation in neuroendocrine neoplasms. Therefore, broader inflammatory evidence is only used in this review to define biological hypotheses and evidence gaps, whereas conclusions regarding GEP-NENs are based on studies directly evaluating neuroendocrine tumour cohorts.

Bulk RNA-sequencing studies in pancreatic neuroendocrine tumours have shown that inflammatory and immune-related pathways may differ according to metastatic status. In metastatic pancreatic neuroendocrine tumours, enrichment of inflammatory response signatures, tumour necrosis factor-α signalling via NF-κB, and IL-6/JAK/STAT3 signalling has been reported, supporting the relevance of cytokine-associated pathways in more aggressive disease phenotypes [180]. These findings are consistent with earlier observations linking STAT3 activation with metastatic disease in GEP-NENs [168]. Nevertheless, such data remain primarily pathway-level associations and do not establish whether IL-6 is produced by tumour cells, stromal elements, infiltrating immune cells, or systemic inflammatory sources [180].

Single-cell RNA sequencing has further demonstrated substantial cellular heterogeneity within GEP-NETs and pancreatic neuroendocrine tumours. Recent studies have identified diverse malignant neuroendocrine cell states as well as distinct immune, myeloid, fibroblast, and endothelial compartments [181,182]. In pancreatic neuroendocrine tumours, single-cell analyses have suggested that genomic instability and histological grade are associated with differences in malignant and immune-cell states, including macrophage-related programmes and inflammatory gene expression [182]. Similarly, the single-cell profiling of small intestinal neuroendocrine tumours has revealed distinct epithelial-like and neuronal-like transcriptional programmes and variable proliferation across malignant and non-malignant cell populations [183]. These findings indicate that cytokine-related signalling may be strongly context-dependent and influenced by tumour site, grade, differentiation status, and microenvironmental composition [181,182,183].

Despite these advances, direct evidence defining IL-1β- or IL-6-producing cellular sources in GEP-NENs remains limited. In particular, it remains unclear whether IL-1β-related activity is mainly associated with inflammasome activation in myeloid cells, tumour-associated macrophages, or tumour cells themselves, and whether IL-6/JAK/STAT3 signalling is predominantly tumour-intrinsic or driven by stromal- and immune-cell interactions. Spatial transcriptomics and multiplex tissue imaging may be especially useful in this context, because they can preserve tissue architecture and clarify whether cytokine-producing cells are located within tumour nests, perivascular regions, invasive fronts, fibrotic stroma, or immune-cell aggregates [183,184].

Proteomic and phosphoproteomic approaches may additionally help determine whether the transcriptional activation of inflammatory pathways translates into functional cytokine signalling. This is particularly relevant for pathways such as JAK/STAT3 and NF-κB, where phosphorylation status, nuclear localization, and spatial proximity between cytokine-producing and cytokine-responsive cells may be more informative than bulk gene expression alone. Future integrative multi-omics studies combining serum biomarkers, tumour transcriptomics, spatial profiling, proteomics, and clinical annotation will be essential to determining whether IL-1β and IL-6 represent causal drivers of GEP-NEN progression, markers of systemic inflammation, or context-dependent components of the neuroendocrine tumour microenvironment [182,184,185].

### 5.7. Therapeutic Implications and Cytokine-Targeted Strategies

The growing recognition of IL-1β and IL-6 as central mediators linking inflammation, angiogenesis, stromal remodelling, immune suppression, and tumour progression has generated considerable interest in cytokine-targeted therapeutic strategies [1,16,40,54,55]. Although direct clinical evidence in GEP-NENs remains limited, several agents targeting the IL-1β/IL-6 signalling axis have demonstrated promising activity in inflammatory diseases and selected malignancies, providing a rationale for their future evaluation in neuroendocrine neoplasms [1,16,40].

Direct therapeutic evidence for IL-1β or IL-6 blockade in GEP-NENs remains scarce. To date, antitumour effects of IL-1β- or IL-6-targeted agents have not been robustly validated in established GEP-NEN cell-line or animal models, and no clinical trial has specifically demonstrated clinical benefit in patients with GEP-NENs. Nevertheless, canakinumab-based IL-1β blockade and tocilizumab-based IL-6R inhibition have been evaluated in early-phase oncology trials, including combinations with chemotherapy or immune checkpoint inhibitors, providing a useful translational framework for future GEP-NEN-directed studies [186,187].

In metastatic pancreatic cancer, IL-1β blockade with canakinumab has been evaluated in combination with PD-1 inhibition and chemotherapy, with reported modulation of systemic myeloid suppression but heterogeneous effects within the tumour microenvironment [186]. In parallel, IL-6R blockade with tocilizumab is being investigated in a phase II trial in combination with ipilimumab and nivolumab in patients with advanced melanoma, non-small-cell lung cancer, or urothelial carcinoma; however, mature efficacy results are not yet available [187]. These studies do not provide direct evidence for GEP-NENs, but they illustrate clinically feasible clinical trial designs combining cytokine blockade with chemotherapy or immune checkpoint inhibition.

Among IL-1 pathway inhibitors, anakinra, a recombinant IL-1 receptor antagonist, blocks signalling mediated by both IL-1α and IL-1β [58]. Anakinra has demonstrated anti-inflammatory and immunomodulatory effects in multiple clinical settings and has been investigated in combination with chemotherapy and immune-based approaches in selected solid tumours. Canakinumab, a monoclonal antibody directed against IL-1β, gained particular attention following the Canakinumab Anti-inflammatory Thrombosis Outcomes Study (CANTOS), which evaluated IL-1β inhibition in patients with atherosclerotic disease [188]. A subsequent cancer-focused analysis of CANTOS unexpectedly demonstrated a reduction in lung cancer incidence and mortality among patients receiving IL-1β blockade [189]. These findings provided proof of concept that targeting chronic inflammation may influence cancer development and progression [58,189].

IL-6-targeted strategies have also attracted substantial interest. Tocilizumab, a monoclonal antibody targeting the IL-6 receptor, is widely used in inflammatory disorders and has been investigated in several malignancies characterized by excessive IL-6 signalling [16,40]. Siltuximab directly neutralizes circulating IL-6, whereas sarilumab inhibits IL-6 receptor signalling. Although these agents have not been systematically evaluated in GEP-NENs, their mechanisms of action may be relevant given the observed associations between IL-6 expression, tumour burden, disease progression, and activation of the JAK/STAT3 pathway in neuroendocrine tumours [158,159,163,170].

Downstream inhibition of cytokine signalling represents an additional therapeutic strategy. Since IL-6 exerts many of its biological effects through JAK/STAT3 activation, JAK inhibitors and emerging STAT3-targeted therapies have been proposed as potential approaches for disrupting tumour-promoting inflammatory circuits [8,48,51]. Experimental studies in several tumour types suggest that inhibition of JAK/STAT signalling may reduce proliferation, angiogenesis, immune suppression, and therapeutic resistance. Whether similar effects may be achieved in GEP-NENs remains unknown and warrants further investigation [170].

Future therapeutic strategies may also involve combination approaches. Given the central role of inflammatory signalling within the tumour microenvironment, cytokine-targeted therapies could theoretically enhance the efficacy of currently established treatments, including somatostatin analogues, peptide receptor radionuclide therapy (PRRT), targeted therapies, and immune checkpoint inhibitors [12,40]. The modulation of IL-1β- and IL-6-dependent pathways may alter immune-cell recruitment, stromal remodelling, angiogenesis, and treatment resistance, thereby increasing susceptibility to other anticancer interventions [40,55].

Despite these promising perspectives, several challenges remain. IL-1β and IL-6 play essential physiological roles in host defence, tissue repair, immune regulation, and neuroendocrine–immune communication; therefore, long-term cytokine blockade may increase susceptibility to infections, impair physiological inflammatory responses, and potentially affect normal neuroendocrine function. Furthermore, the biological heterogeneity of GEP-NENs suggests that only selected patient subsets may benefit from cytokine-targeted therapies [12,18,158,163]. The identification of predictive biomarkers, clarification of tumour-intrinsic versus microenvironment-driven cytokine signalling, and validation of clinically relevant therapeutic targets remain major priorities for future translational research [16,40].

Therapeutic strategies targeting IL-1β-, IL-6-, and JAK/STAT-related pathways that may be relevant to GEP-NENs are summarized in Table 5.

### 5.8. IL-6 in the Context of Established Biomarkers

Although IL-6 has emerged as one of the most promising inflammatory biomarkers in GEP-NENs, its potential clinical utility should be evaluated in the context of currently established biomarkers and diagnostic modalities. At present, chromogranin A (CgA), NETest, Ki-67 proliferation index, neuron-specific enolase (NSE), and molecular imaging techniques remain the most widely used tools for disease characterization, prognostication, and treatment monitoring in neuroendocrine neoplasms.

CgA remains the most commonly used circulating biomarker in clinical practice; however, its diagnostic and prognostic performance is limited by variable sensitivity, reduced specificity, and susceptibility to multiple confounding factors, including proton pump inhibitor use, renal dysfunction, and chronic inflammatory conditions [12]. In contrast, IL-6 may reflect a different aspect of tumour biology, namely tumour-associated inflammation and host immune responses. Consequently, IL-6 should not be viewed as a replacement for CgA but rather as a potentially complementary biomarker.

An important limitation of circulating IL-6 measurements is their relatively low disease specificity. Unlike tumour-specific biomarkers, elevated serum IL-6 concentrations may result from a variety of non-neoplastic conditions, including obesity, type 2 diabetes mellitus, metabolic syndrome, chronic inflammatory disorders, autoimmune diseases, and acute infections [16,40,58]. These factors may act as important confounders when interpreting circulating IL-6 levels in patients with GEP-NENs, particularly given the frequent coexistence of metabolic comorbidities in this patient population. Consequently, elevated IL-6 concentrations should not be interpreted as direct evidence of tumour progression without consideration of concurrent inflammatory or metabolic conditions.

This limitation may partially explain the variability observed across published studies evaluating IL-6 in neuroendocrine neoplasms. Future biomarker studies should therefore incorporate careful assessment of metabolic and inflammatory comorbidities and determine whether IL-6 provides clinically relevant information independent of these confounding factors.

NETest represents a more comprehensive liquid-biopsy approach based on multigene transcriptomic analysis and has demonstrated superior diagnostic accuracy compared with conventional circulating biomarkers in several studies [12]. Nevertheless, NETest primarily reflects tumour-specific gene expression patterns, whereas IL-6 may provide additional information regarding inflammatory activity within the tumour microenvironment and systemic host responses. Whether combining NETest with inflammatory biomarkers improves risk stratification remains unknown.

Ki-67 remains the most important pathological marker for tumour grading and prognostication in GEP-NENs [160]. Unlike Ki-67, which reflects proliferative activity at a single tissue sampling site, circulating IL-6 concentrations may potentially capture dynamic systemic changes associated with tumour progression, metastatic dissemination, and inflammatory remodelling. However, current evidence remains insufficient to determine whether IL-6 provides prognostic information independent of tumour grade.

Similarly, NSE is primarily used in poorly differentiated neuroendocrine neoplasms and is generally regarded as a marker of tumour burden and aggressive disease [12]. The potential relationship between NSE and IL-6 has not been systematically investigated; comparative studies remain lacking.

Molecular imaging techniques, including somatostatin receptor imaging with gallium-68-labelled DOTATATE positron emission tomography/computed tomography (68Ga-DOTATATE PET/CT) and fluorine-18 fluorodeoxyglucose positron emission tomography/computed tomography (18F-FDG PET/CT), provide information regarding receptor expression, tumour differentiation, metabolic activity, and disease extent [12]. These imaging approaches currently offer substantially greater clinical utility than any circulating inflammatory biomarker. However, imaging studies do not directly characterize inflammatory signalling pathways. Therefore, IL-6 may provide complementary biological information that cannot be obtained through imaging alone.

Overall, the currently available evidence does not support the use of IL-6 as a standalone diagnostic or prognostic biomarker in GEP-NENs. Instead, its greatest potential may lie in multimodal biomarker strategies integrating tumour-specific markers, histopathological parameters, molecular imaging findings, and inflammatory profiling. Prospective studies directly comparing IL-6 with established biomarkers and evaluating their combined performance are needed before clinical implementation can be considered.

The potential complementary role of IL-6 in relation to established biomarkers and diagnostic modalities in GEP-NENs is summarized in Table 6.

### 5.9. IL-1β and IL-6 Across Tumour Grade and Disease Advancement

From a clinical perspective, an important question is whether IL-1β and IL-6 expression differs across tumour differentiation and grade, particularly between well-differentiated NET G1/G2, well-differentiated NET G3, and poorly differentiated NECs. This distinction is clinically relevant because these entities differ markedly in prognosis, molecular background, proliferative activity, and therapeutic strategy.

The strongest currently available evidence concerns IL-6. Herman Mahečić et al. reported high IL-6 expression in GEP-NEN tissue samples and observed that IL-6 expression tended to increase with higher tumour grade, although this association did not reach statistical significance [159]. Serum-based studies have similarly associated elevated IL-6 concentrations with more advanced disease characteristics. Geisler et al. reported a significant correlation between serum IL-6 concentrations and tumour stage in patients with well-differentiated gastrointestinal neuroendocrine tumours [158]. Pereira et al. additionally observed that increased IL-6 expression in tumour tissue correlated with disease progression [178].

Collectively, these findings suggest that IL-6 may be associated with more advanced or biologically aggressive GEP-NEN phenotypes. The biological basis for higher IL-6 expression in more aggressive GEP-NEN phenotypes is likely multifactorial. Poorly differentiated or high-grade tumours are characterized by higher proliferative activity, genomic instability, hypoxia, necrosis, and more pronounced tissue damage, all of which may promote the activation of NF-κB- and STAT3-dependent inflammatory programmes within tumour-, stromal-, and immune-cell compartments. In this setting, IL-6 may not only reflect tumour-cell intrinsic signalling, but also microenvironmental activation, macrophage and fibroblast recruitment, angiogenic remodelling, and systemic inflammatory responses associated with aggressive disease. Therefore, differences in IL-6 expression between indolent NET G1/G2 and high-grade NECs should currently be interpreted as a marker of broader inflammatory and biologically aggressive tumour ecosystems rather than as an NEC-specific mechanism. Direct comparative studies separating well-differentiated NET G3 from poorly differentiated NECs are still required. In contrast, evidence regarding IL-1β across tumour grade and disease advancement remains less clearly defined. Although IL-1β expression has been detected in tumour tissue and elevated serum concentrations have been reported in patients with NENs, currently available data do not clearly establish relationships between IL-1β and tumour differentiation, proliferative activity, or progression from NET to NEC.

Overall, IL-6 currently appears to be the more consistently reported cytokine in relation to tumour grade, stage, and disease progression, whereas the role of IL-1β in this context remains predominantly exploratory and biologically oriented.

### 5.10. Diagnostic, Prognostic, and Biological Significance

The available evidence suggests three principal interpretations of IL-1β and IL-6 in GEP-NENs: diagnostic, prognostic, and biological. These potential roles should be clearly distinguished.

First, IL-1β and IL-6 may have diagnostic relevance, as several studies have demonstrated elevated serum concentrations in patients with NENs compared with healthy controls [158,163,177]. However, both cytokines represent non-specific inflammatory mediators, and their elevation may therefore reflect systemic inflammatory activity rather than GEP-NEN-specific biology. At present, neither IL-1β nor IL-6 can be regarded as validated diagnostic biomarkers for GEP-NENs.

Second, IL-6 appears to be the more consistently reported prognostic candidate. Associations between IL-6 and tumour stage, tumour grade, or disease progression have been observed in both serum- and tissue-based studies [158,159,178]. These findings suggest that IL-6 may reflect tumour burden or biologically aggressive disease features and may potentially complement established clinicopathological prognostic parameters.

Third, IL-1β and IL-6 may serve as biological markers reflecting inflammatory activation within the tumour and the surrounding tumour microenvironment (TME). This interpretation currently appears particularly plausible. Increased cytokine expression in tumour tissue and correlations with chromogranin A concentrations support the concept that these cytokines may participate in, or reflect, inflammatory remodelling processes in GEP-NENs [159,163].

Taken together, IL-6 may currently be interpreted as a candidate marker of inflammatory activity and disease aggressiveness, whereas IL-1β appears to be more closely associated with inflammatory signalling and potential susceptibility mechanisms. Nevertheless, neither cytokine has entered routine clinical decision-making in GEP-NENs.

### 5.11. Translational Implications: Biologically Attractive but Not Yet Clinically Actionable

IL-1β and IL-6 represent biologically attractive therapeutic targets because they link chronic inflammation with immune regulation, stromal activation, angiogenesis, and tumour progression. In broader oncology, blockade of IL-1- and IL-6-related pathways has been explored as a strategy to reduce cancer-associated inflammation, modulate the TME, and potentially improve treatment responses.

These concepts are also relevant to GEP-NENs, in which inflammatory cytokines may influence tumour–stromal interactions, systemic inflammatory activity, immune escape, and disease progression. However, direct evidence defining CAF-derived IL-1β or IL-6 signalling in GEP-NENs remains limited, and most mechanistic insights are currently extrapolated from pancreatic adenocarcinoma and other solid tumour models.

Despite this biological rationale, the translation of these observations into clinical management of GEP-NENs remains at an early stage. At present, there is insufficient evidence to support routine therapeutic targeting of IL-1β or IL-6 in patients with GEP-NENs. Importantly, no interventional studies have yet demonstrated whether a blockade of IL-1β- or IL-6-related pathways provides a clinically meaningful benefit in this tumour group. It also remains unclear whether the modulation of these pathways could improve tumour control, symptom control, progression-free survival, overall survival, or responsiveness to established therapies, including somatostatin analogues, peptide receptor radionuclide therapy (PRRT), targeted therapies, chemotherapy, or immunotherapy.

Future translational studies may clarify whether IL-1β or IL-6 identify biologically distinct GEP-NEN subgroups, predict therapeutic response, or represent rational combination targets alongside currently available treatment strategies.

### 5.12. Critical Synthesis of the Available Evidence

Overall, the available studies suggest that IL-1β and IL-6 may contribute to the inflammatory biology of GEP-NENs, although the strength of the evidence differs substantially between these cytokines. IL-6 is the more consistently reported mediator, with associations observed in relation to serum concentrations, tissue expression, tumour stage, tumour grade, and disease progression. Its role appears particularly relevant in the context of systemic inflammatory activity, JAK/STAT3 signalling, tumour aggressiveness, and possibly non-functioning pancreatic NENs.

The recent GEP-NEN-focused literature on the tumour microenvironment strengthens the view that IL-1β and IL-6 should not only be evaluated as isolated circulating cytokines, but as components of broader inflammatory networks involving stromal composition, immune-cell infiltration, vascular remodelling, extracellular matrix organization, and treatment exposure. This perspective may help clarify whether these cytokines identify biologically distinct GEP-NEN subgroups, reflect tumour–stroma interactions, or contribute to resistance to established treatments. However, these remain translational hypotheses that require validation in neuroendocrine tumour-specific cohorts.

IL-1β is likewise biologically plausible, particularly through its relationship with NF-κB activation, inflammatory amplification, and genetic susceptibility. However, its specific clinical role in GEP-NENs remains less clearly defined. Current data suggest that IL-1β may contribute to inflammatory signalling and may be associated with susceptibility to pancreatic NENs in selected genetic studies, although evidence supporting its prognostic or therapeutic significance remains less consistent than that available for IL-6.

Accordingly, IL-6 may currently be regarded as a promising exploratory marker associated with inflammatory activation, tumour burden, and aggressive disease characteristics, whereas IL-1β remains a biologically relevant but less clinically defined mediator. Both cytokines warrant further investigation, particularly in studies integrating serum cytokine profiles, tissue expression, genetic background, tumour grade, primary tumour localization, functional status, treatment response, and clinical outcomes.

Importantly, the currently available evidence regarding IL-1β and IL-6 in GEP-NENs remains preliminary and should therefore be interpreted with caution. Most studies are based on relatively small cohorts and frequently combine tumours of different primary sites, functional status, grades, and stages, thereby limiting conclusions regarding clinically relevant subgroups such as pancreatic versus gastrointestinal NENs, functioning versus non-functioning tumours, NETs versus NECs, and localized versus metastatic disease. Additional limitations include potential selection bias, heterogeneity of analytical platforms, lack of standardized cut-off values, insufficient adjustment for confounding variables, limited external validation, and absence of interventional studies. Consequently, the current evidence should primarily be regarded as exploratory and hypothesis-generating rather than sufficient to establish IL-1β or IL-6 as validated diagnostic, prognostic, or therapeutic biomarkers in GEP-NENs.

Table 7 summarizes original studies evaluating associations between IL-1β and/or IL-6 and GEP-NENs.

NENs represent a clinically challenging group of tumours. Despite advances in diagnostic modalities, including improved availability of endoscopic procedures and modern imaging techniques, diagnosis is frequently delayed. Marked biological heterogeneity and non-specific clinical manifestations often contribute to substantial diagnostic difficulty [190]. Data from an international study demonstrated that the mean interval between the onset of initial symptoms and the diagnosis of NENs was approximately 52 months [191].

Consequently, increasing attention has focused on the identification of novel biomarkers that could improve diagnostic accuracy and support the monitoring of disease course and treatment response. Growing evidence suggests that inflammatory mediators, including IL-1β and IL-6, may reflect TME dynamics and disease activity; however, their potential clinical utility in GEP-NENs requires further investigation and prospective validation.

## 6. Conclusions

Inflammatory cytokines, particularly IL-1β and IL-6, represent important molecular links between chronic inflammation, tumour progression, remodelling of the TME, immune modulation, angiogenesis, and therapeutic resistance. Through NF-κB- and STAT3-dependent signalling, these cytokines contribute to tumour-promoting inflammatory circuits across multiple cancer types.

In GEP-NENs, currently available evidence suggests that IL-6 may be associated with systemic inflammatory activity, tumour burden, tumour grade, and disease progression, whereas IL-1β appears to be more closely related to local inflammatory signalling, microenvironmental remodelling, and potential susceptibility mechanisms. Nevertheless, these observations should be regarded as exploratory and hypothesis-generating rather than definitive.

Importantly, available evidence remains limited by relatively small patient cohorts, heterogeneous tumour populations, variable analytical methodologies, the lack of standardized cut-off values, and the absence of prospective validation studies. Consequently, neither IL-1β nor IL-6 can currently be considered validated diagnostic, prognostic, or therapeutic biomarkers in GEP-NENs, and routine therapeutic targeting of these pathways is not supported by existing clinical evidence.

At present, the strongest evidence supports the biological and translational relevance of IL-1β- and IL-6-related signalling rather than immediate clinical applicability. These cytokines may contribute to inflammatory remodelling of the TME and may reflect aggressive tumour behaviour; however, their precise role in GEP-NEN pathobiology and clinical management remains incompletely understood.

Future studies should evaluate IL-1β and IL-6 in larger, well-characterized cohorts stratified according to primary tumour site, differentiation status, grade, functional status, disease stage, and treatment exposure. The integration of serum cytokine profiles with tissue expression patterns, genetic background, and established clinicopathological parameters may help determine whether these cytokines can contribute to clinically meaningful risk stratification, biomarker development, or future translational therapeutic strategies in GEP-NENs.

Importantly, the available evidence predominantly derives from studies involving well-differentiated gastroenteropancreatic neuroendocrine tumours, whereas data regarding poorly differentiated neuroendocrine carcinomas remain limited. Consequently, direct extrapolation of cytokine-related findings across all neuroendocrine neoplasm subtypes should be approached with caution.

## Figures and Tables

**Table 1:** Selected original studies on the role of IL-1β in carcinogenesis.

Research	Cancer Type	Study Design/Model	Key IL-1β Findings	Main Limitation	References
Caronni N. et al.	Pancreatic ductal adenocarcinoma	Translational single-cell/spatial genomics study with in vitro and in vivo validation.	IL-1β-expressing TAMs promoted inflammatory reprogramming of PDAC cells and were associated with disease progression. Blockade of PGE2 or IL-1β reduced tumour-promoting inflammation and improved PDAC control in vivo.	PDAC-specific mechanistic findings requiring further clinical validation.	[87]
Takahashi R. et al.	Pancreatic ductal adenocarcinoma	Preclinical/translational study using KC-IL1β mouse models, pancreatic 3D cultures, and human PDAC samples.	IL-1β-induced pancreatitis accelerated PDAC progression and liver metastasis while promoting immunosuppressive PD-L1^+^ regulatory B-cell expansion.	Primarily mouse-model based; clinical relevance remains unconfirmed.	[88]
Spagnardi M. et al.	Colorectal cancer	Preclinical in vitro study using colorectal cancer cell lines.	IL-1β reduced 5-FU sensitivity, activated NF-κB signalling, and promoted IL-8 secretion, supporting a role in CRC chemoresistance.	Cell-line study lacking in vivo and clinical validation.	[89]
Li Y. et al.	Colorectal cancer	Preclinical in vitro study using colon cancer cell lines and primary human cells.	IL-1β promoted EMT, stemness-associated features, invasion, and carboplatin resistance through the IL-1β–Zeb1–Bmi1 axis.	Mainly in vitro findings with limited primary patient-derived material.	[90]
Gao Z. et al.	Hepatocellular carcinoma	Translational study combining HCC tissue analyses, macrophage–tumour co-culture models, and mouse metastasis models.	Macrophage autophagy inhibition enhanced IL-1β release and promoted EMT, invasion, and metastasis in HCC. IL-1β blockade reduced lung metastasis in vivo.	Predominantly mechanistic/preclinical observations requiring prospective validation.	[91]
Sheikhhossein H.H. et al.	Non-small-cell lung cancer	Preclinical/translational study using EGFR-mutant NSCLC models and tumour-derived extracellular vesicles.	IL-1β promoted EMT-related signalling, extracellular vesicle-mediated communication, and immunomodulatory changes in NSCLC.	Focused on selected EGFR-mutant NSCLC models with limited patient validation.	[92]
Nisar M.A. et al.	Breast cancer	Preclinical study using breast cancer cell lines and mouse xenografts.	IL-1β promoted vasculogenic mimicry and activated p38/MAPK and PI3K/Akt signalling pathways associated with breast cancer aggressiveness.	Predominantly cell-line based with limited in vivo validation.	[93]
Storr S.J. et al.	Breast cancer	Preclinical in vitro study using breast cancer and endothelial cell models.	IL-1β enhanced tumour cell adhesion, migration, and transmigration, supporting a role in lymphovascular invasion and metastatic dissemination.	In vitro model lacking clinical and in vivo confirmation.	[94]
Hu H. et al.	Ovarian cancer	Translational study using ovarian cancer tissues, cell lines, MDSC co-cultures, and mouse models.	An SAA1–MDSC–IL-1β feedback loop promoted immunosuppression, tumour progression, immune evasion, and ascites formation.	Complex mechanistic study requiring prospective clinical validation.	[95]
Chang M.A. et al.	Prostate cancer	Preclinical in vitro study using prostate cancer cell lines.	IL-1β suppressed androgen receptor signalling and promoted treatment-resistant phenotypes in prostate cancer cells.	In vitro observations requiring validation in clinical cohorts.	[96]

Abbreviations: 3D, three-dimensional; 5-FU, 5-fluorouracil; Akt, protein kinase B; Bmi1, B-cell-specific Moloney murine leukemia virus integration site 1; CRC, colorectal cancer; EGFR, epidermal growth factor receptor; EMT, epithelial–mesenchymal transition; HCC, hepatocellular carcinoma; IL-1β, interleukin-1 beta; IL-8, interleukin-8; KC-IL1β, Kras-mutant mouse model with IL-1β overexpression; MAPK, mitogen-activated protein kinase; MDSC, myeloid-derived suppressor cell; NF-κB, nuclear factor kappa B; NSCLC, non-small-cell lung cancer; PDAC, pancreatic ductal adenocarcinoma; PD-L1, programmed death-ligand 1; PGE2, prostaglandin E2; PI3K, phosphoinositide 3-kinase; SAA1, serum amyloid A1; TAMs, tumour-associated macrophages; VEGFR-1, vascular endothelial growth factor receptor 1; Zeb1, zinc finger E-box-binding homeobox 1.

**Table 2:** Selected original studies on the role of IL-6 in carcinogenesis.

Research	Cancer Type	Study Design/Model	Key IL-6-Related Findings	Main Limitation	References
Zhang Y. et al.	Pancreatic cancer	Preclinical in vitro study using pancreatic cancer tissues and cell models.	IL-6 promoted proliferation, EMT, migration, and metastatic features through the IL-6/miR-455-5p/IGF-1R axis in pancreatic cancer cells.	Predominantly preclinical findings requiring clinical validation.	[148]
Huang Y.H. et al.	Colorectal cancer	Preclinical in vitro study using colorectal cancer cell models.	IL-6/sIL-6R induced EMT through Src/FAK-, STAT3-, and NF-κB-dependent signalling, supporting a role for IL-6 in CRC invasion and migration.	Cell-line study without in vivo or clinical validation.	[149]
Han J. et al.	Colorectal cancer	Preclinical/translational study using a mouse model of colitis-associated colorectal cancer and human CAC samples.	IL-6 receptor blockade reduced colorectal neoplasm formation and downregulated HIF-1α expression, supporting the role of the IL-6/HIF-1α axis in inflammation-associated carcinogenesis.	Limited human cohort and predominantly experimental model.	[150]
De Simone V. et al.	Colorectal cancer	Translational study using CRC patient-derived immune cells, CRC cell lines, and Apcmin/+ mouse models.	IL-6 contributed to STAT3/NF-κB-dependent CRC proliferation and tumour-promoting cytokine signalling within the TME.	IL-6 acted within a redundant cytokine network, limiting interpretation of IL-6-specific effects.	[151]
Wu X. et al.	Gastric cancer	Translational study using gastric cancer tissues, CAFs, and mouse metastasis models.	CAF-derived IL-6 promoted EMT, migration, and peritoneal metastasis through JAK2/STAT3 signalling in gastric cancer.	Mechanistic study lacking prospective clinical confirmation.	[152]
Chan L.C. et al.	Hepatocellular carcinoma	Translational/mechanistic study using HCC models and immunotherapy mouse models.	IL-6 promoted PD-L1 glycosylation and immune evasion through the IL-6/JAK1/PD-L1/STT3A axis in HCC.	Complex mechanistic study lacking prospective clinical confirmation.	[153]
Liu J. et al.	Non-small-cell lung cancer	Preclinical/translational study using NSCLC patient samples, cell lines, and mouse models.	IL-6 induced FGL1 expression through STAT3 activation, promoting EMT, migration, and tumour progression in NSCLC.	Limited patient validation and no prospective clinical confirmation.	[154]
Shang G.S. et al.	Non-small-cell lung cancer	Clinical observational study using NSCLC tumour and serum samples.	Elevated IL-6 levels correlated with EMT markers and metastatic disease in NSCLC.	Observational design without mechanistic validation.	[155]
Zhao C. et al.	Breast cancer	Preclinical/translational study using breast cancer specimens, co-culture systems, and mouse models.	Adipocyte-derived IL-6 promoted EMT, metastasis, and PD-L1 expression through STAT3-dependent signalling in breast cancer.	Mainly mechanistic/preclinical findings without clinical interventional validation.	[156]
Zhang T. et al.	Ovarian cancer	Preclinical/translational study using ovarian cancer cell lines, xenografts, and TCGA analyses.	IL-6 and hypoxia synergistically promoted EMT and invasion through the IL-6/STAT3/HIF-1α feedback loop.	Limited in vivo validation and lack of clinical confirmation.	[157]

Abbreviations: Apcmin/+, adenomatous polyposis coli multiple intestinal neoplasia mouse model; CAC, colitis-associated colorectal cancer; CAFs, cancer-associated fibroblasts; CRC, colorectal cancer; EMT, epithelial–mesenchymal transition; FAK, focal adhesion kinase; FGL1, fibrinogen-like protein 1; HCC, hepatocellular carcinoma; HIF-1α, hypoxia-inducible factor 1-alpha; IGF-1R, insulin-like growth factor 1 receptor; IL-6, interleukin-6; IL-6R, interleukin-6 receptor; JAK1, Janus kinase 1; JAK2, Janus kinase 2; miR-455-5p, microRNA-455-5p; NF-κB, nuclear factor kappa B; NSCLC, non-small-cell lung cancer; PD-L1, programmed death-ligand 1; sIL-6R, soluble interleukin-6 receptor; Src, SRC proto-oncogene, non-receptor tyrosine kinase; STAT3, signal transducer and activator of transcription 3; STT3A, STT3 oligosaccharyltransferase complex catalytic subunit A; TCGA, The Cancer Genome Atlas; TME, tumour microenvironment.

**Table 3:** Potential differences in IL-1β and IL-6 signalling across the spectrum of GEP-NENs.

Feature	Well-Differentiated NETs (G1/G2)	NET G3	Poorly Differentiated NECs
Biological behaviour	Indolent or slowly progressive	Intermediate aggressiveness	Highly aggressive
Ki-67 proliferation index	Low to moderate	Elevated	Markedly elevated
Tumour microenvironment	Relatively less inflammatory	Intermediate inflammatory activity	Highly inflammatory and immunologically active
Hypoxia and necrosis	Usually limited	More frequent	Common
Stromal remodelling	Moderate	Increased	Extensive
NF-κB activation	Potentially present but incompletely characterized	Likely increased	Expected to be strongly activated
STAT3 activation	Reported in some tumours	Potentially increased	Likely enhanced
IL-1β expression	Variable; limited available evidence	Insufficient data	Presumed increased, but direct evidence lacking
IL-6 expression	Frequently elevated compared with controls	Higher expression reported in higher-grade tumours	Expected to be highest, although dedicated comparative studies remain limited
Association with tumour progression	Uncertain	Emerging evidence	Likely relevant
Current strength of evidence	Moderate	Limited	Very limited
Main limitation of available studies	Small cohorts and heterogeneous populations	Lack of dedicated studies	Frequent pooling with NETs in published analyses

Abbreviations: G1, grade 1; G2, grade 2; G3, grade 3; GEP-NENs, gastroenteropancreatic neuroendocrine neoplasms; IL-1β, interleukin-1 beta; IL-6, interleukin-6; NET, neuroendocrine tumour; NEC, neuroendocrine carcinoma; NF-κB, nuclear factor kappa B; STAT3, signal transducer and activator of transcription 3. Note: The table summarizes published observations and biologically plausible mechanisms derived from both GEP-NEN-specific studies and the broader oncology literature. Direct comparative studies separating well-differentiated NETs, NET G3, and poorly differentiated NECs remain limited; therefore, the proposed relationships should be interpreted as hypothesis-generating rather than definitive.

**Table 4:** Comparative Overview of IL-1β and IL-6 in Gastroenteropancreatic Neuroendocrine Neoplasms (GEP-NENs).

Feature	IL-1β	IL-6
Main biological function	Initiation and amplification of inflammatory responses; inflammasome-dependent signalling	Regulation of chronic inflammation, tumour progression, angiogenesis, and immune modulation
Principal signalling pathways	NLRP3 inflammasome, IL-1R/MyD88/NF-κB	IL-6R/gp130/JAK/STAT3, NF-κB
Evidence in GEP-NENs	Limited	Moderate
Serum biomarker studies	Few available studies	Multiple studies available
Tissue expression studies	Reported mainly in pancreatic NENs	Reported in both pancreatic and gastrointestinal NENs
Association with tumour grade	Inconsistent evidence	Positive association reported in several studies
Association with metastatic disease	Limited data	Frequently associated with advanced disease and tumour burden
Prognostic significance	Not yet established	Potential prognostic value suggested
Tumour microenvironment involvement	Likely participates in immune-cell recruitment and inflammatory activation	Involved in stromal remodelling, angiogenesis, immune suppression, and tumour progression
NET versus NEC differences	Poorly characterized	Emerging evidence suggests higher expression in aggressive tumours
Functional versus non-functional tumours	Limited evidence	Differences reported in selected studies
Therapeutic targeting	Anakinra, canakinumab, other IL-1 pathway inhibitors	Tocilizumab, siltuximab, sarilumab, JAK/STAT inhibitors
Current translational potential	Exploratory	More advanced
Major limitation of current evidence	Small number of studies and limited validation	Lack of prospective validation and clinical utility studies
Overall strength of evidence in GEP-NENs	Low	Moderate

Abbreviations: GEP-NENs, gastroenteropancreatic neuroendocrine neoplasms; gp130, glycoprotein 130; IL-1β, interleukin-1 beta; IL-1R, interleukin-1 receptor; IL-6, interleukin-6; IL-6R, interleukin-6 receptor; JAK, Janus kinase; MyD88, myeloid differentiation primary response 88; NECs, neuroendocrine carcinomas; NENs, neuroendocrine neoplasms; NETs, neuroendocrine tumours; NF-κB, nuclear factor kappa B; NLRP3, NOD-like receptor family pyrin domain-containing 3; STAT3, signal transducer and activator of transcription 3.

**Table 5:** Cytokine-targeted therapeutic strategies relevant to GEP-NENs.

Therapeutic Target	Representative Agents	Mechanism of Action	Current Clinical Status	Potential Relevance to GEP-NENs
IL-1β	Canakinumab	Neutralization of IL-1β	Approved for inflammatory disorders; oncology interest following CANTOS	Potential reduction in tumour-promoting inflammation
IL-1R	Anakinra	IL-1 receptor blockade	Approved for inflammatory diseases; investigated in oncology	Modulation of inflammasome-related signalling
IL-6	Siltuximab	Direct IL-6 neutralization	Approved for Castleman disease	Potential suppression of IL-6-dependent tumour progression
IL-6R	Tocilizumab	IL-6 receptor blockade	Approved for autoimmune disorders and CRS	Potential inhibition of IL-6/JAK/STAT3 signalling
IL-6R	Sarilumab	IL-6 receptor blockade	Approved for rheumatoid arthritis	Potential modulation of chronic inflammatory signalling
JAK	Ruxolitinib and other JAK inhibitors	Inhibition of JAK-mediated cytokine signalling	Approved in hematologic malignancies	Potential disruption of downstream IL-6 signalling
STAT3	Experimental STAT3 inhibitors	Direct inhibition of STAT3 activity	Early-phase development	Potential suppression of tumour growth, angiogenesis, and immune evasion

Abbreviations: CANTOS, Canakinumab Anti-inflammatory Thrombosis Outcomes Study; CRS, cytokine release syndrome; GEP-NENs, gastroenteropancreatic neuroendocrine neoplasms; IL-1β, interleukin-1 beta; IL-1R, interleukin-1 receptor; IL-6, interleukin-6; IL-6R, interleukin-6 receptor; JAK, Janus kinase; STAT3, signal transducer and activator of transcription 3.

**Table 6:** Comparison of IL-6 with established biomarkers and diagnostic modalities in GEP-NENs.

Biomarker/Modality	Main Information Provided	Strengths	Limitations	Potential Relationship with IL-6
Chromogranin A (CgA)	Tumour burden and neuroendocrine differentiation	Widely available	Limited specificity; affected by PPIs and renal dysfunction	IL-6 may provide complementary inflammatory information
NETest	Tumour-specific transcriptomic activity	High diagnostic accuracy	Limited availability and cost	Potentially complementary
Ki-67	Tumour proliferation and grading	Established prognostic marker	Requires tissue sampling	IL-6 may reflect systemic disease activity beyond proliferation
Neuron-specific enolase (NSE)	Aggressive disease biology	Useful in NECs	Limited sensitivity in well-differentiated NETs	Relationship remains poorly defined
68Ga-DOTATATE PET/CT	Somatostatin receptor expression	Excellent staging and treatment selection	Does not assess inflammatory signalling	IL-6 may provide additional biological context
18F-FDG PET/CT	Tumour metabolism and aggressiveness	Useful in higher-grade disease	Limited sensitivity in indolent NETs	Potential association with inflammatory activation
IL-6	Tumour-associated inflammation and host response	Non-invasive; biologically informative	Limited validation and specificity	Potential complementary biomarker

Abbreviations: 18F-FDG, fluorine-18 fluorodeoxyglucose; 68Ga-DOTATATE, gallium-68-labelled DOTATATE; CgA, chromogranin A; GEP-NENs, gastroenteropancreatic neuroendocrine neoplasms; IL-6, interleukin-6; NECs, neuroendocrine carcinomas; NETest, multigene neuroendocrine transcriptomic test; NETs, neuroendocrine tumours; NSE, neuron-specific enolase; PET/CT, positron emission tomography/computed tomography; PPIs, proton pump inhibitors.

**Table 7:** Studies Evaluating IL-1β and IL-6 in GEP-NENs.

Study	Sample Size	Tumour Site/ Subgroup	WHO Grade/ Differentiation	Specimen Type	IL-1β/ IL-6 Assay	Endpoint	Main Conclusion	Prognostic Significance/Limitation	References
Budek M. et al.	84 well-differentiated NET patients; 40 healthy controls	Mixed NET cohort including pancreatic NETs (*n* = 22), gastrointestinal NETs (*n* = 32), lung NETs (*n* = 12), and NETs of other/unclassified sites (*n* = 18)	Well-differentiated NET G1/G2 only; G1 64%, G2 36%; primary tumours without evident metastatic disease; NECs were not included	Serum	Multiplex ELISA/Bio-Plex Pro Human Cytokine Screening Panel for 48 inflammatory cytokines, including IL-1β and IL-6; CgA measured by ELISA	Diagnostic performance of circulating cytokines and correlation with CgA	Serum IL-1β and IL-6 were significantly elevated in NET patients compared with healthy controls; IL-6 showed a weak positive correlation with CgA.	Findings suggest possible diagnostic or monitoring relevance of circulating inflammatory cytokines in well-differentiated NETs, but they should not be extrapolated to poorly differentiated NECs; GEP-specific conclusions are limited by the mixed tumour-site cohort, absence of tissue validation, and lack of direct prognostic endpoints.	[163]
Strzelczyk J. et al.	80 NET patients; 62 healthy controls	Mixed NET cohort including GEP-NETs (*n* = 64: pancreatic *n* = 24, small intestinal *n* = 21, duodenal *n* = 4, gastric *n* = 8, rectal *n* = 7) and BP-NETs (*n* = 16: typical carcinoids *n* = 10, atypical carcinoids *n* = 6)	NET cohort classified according to WHO 2019 for GEP-NETs and WHO 2015 for BP-NETs; grading in the whole cohort: G1 *n* = 40, G2 *n* = 21, G3 *n* = 3; Ki-67 < 3% *n* = 45, 3–20% *n* = 31, >21% *n* = 4; poorly differentiated NECs were not analyzed as a separate group	Serum	ELISA measurement of IL-6, oncostatin M, and cardiotrophin-1	Comparison of circulating IL-6 family cytokines between NET patients and healthy controls; assessment of associations with Ki-67, clinical stage, primary site, CgA, serotonin, and 5-HIAA	Serum IL-6 was significantly higher in NET patients than in controls, whereas OSM and CT1 were significantly lower. IL-6 did not correlate with Ki-67, clinical stage, primary site, CgA, serotonin, or 5-HIAA.	The study suggests possible diagnostic relevance of circulating IL-6 family cytokines in NETs, but IL-6 showed no clear prognostic association. Findings should not be extrapolated to poorly differentiated NECs; limitations include retrospective single-centre design, mixed GEP-NET/BP-NET cohort, relatively small sample size, and lack of tissue validation.	[177]
Geisler L. et al.	43 well-differentiated GEP-NET patients; 40 healthy controls.	GEP-NET cohort limited to pancreatic NETs (*n* = 20) and ileal NETs (*n* = 23)	Well-differentiated NET G1/G2 only; G1 *n* = 14, G2 *n* = 29; no NECs were included; most patients had advanced disease, including TNM stage IV in 31/43 and distant metastases in 34/43	Serum	Multiplex bead-based cytokine assay using LEGENDplex Human Inflammation Panel 1, including IL-1β and IL-6; CgA measured by TRACE; NETest assessed by quantitative PCR	Diagnostic discrimination between GEP-NET patients and controls; association with tumour grade, primary site, metastatic disease, and disease progression during longitudinal follow-up	In well-differentiated GEP-NET G1/G2 patients, serum IL-1β and IL-6 were significantly elevated compared with healthy controls. IL-6 levels differed significantly between G1 and G2 NETs, with lower IL-6 levels in G2 than G1 tumours; cytokine levels did not differ by pancreatic versus ileal primary site.	The study suggests diagnostic and monitoring relevance of selected serum cytokines in well-differentiated pancreatic/ileal GEP-NETs, but findings should not be extrapolated to NET G3 or poorly differentiated NECs. Baseline cytokine levels did not predict disease progression; longitudinal IL-1β/IL-8 dynamics and IL-10 ratios may reflect disease course. Limitations include retrospective single-centre design, small cohort size, restriction to G1/G2 pancreatic/ileal NETs, and lack of tissue validation.	[158]
Herman Mahečić D. et al.	43 GEP-NEN patients	Gastrointestinal GEP-NEN cohort including gastric (*n* = 14), duodenal (*n* = 1), ileal (*n* = 12), appendiceal (*n* = 12), and colonic (*n* = 4) tumours	GEP-NENs graded according to WHO 2010: G1 *n* = 22, G2 *n* = 14, G3 *n* = 7; NET versus NEC differentiation status was not explicitly separated	Formalin-fixed paraffin-embedded tumour tissue	Immunohistochemistry for TNF-α, IL-1β, IL-2, and IL-6; IL-1β, IL-2, and IL-6 assessed as cytoplasmic staining	Association of tissue cytokine expression with Ki-67 proliferation index, tumour grade, TNM status, and clinical outcome	Most GEP-NENs showed high IL-6 expression and lower IL-1β and IL-2 expression. IL-6 expression increased with tumour grade, although this trend did not reach statistical significance. TNF-α, rather than IL-6 or IL-1β, was significantly associated with higher Ki-67, higher tumour grade, and death outcome.	The study supports local inflammatory cytokine expression in GEP-NEN tissue, but IL-6 and IL-1β were not independently validated as prognostic markers. Conclusions regarding NET versus NEC should be limited because differentiation status of G3 cases was not explicitly separated; limitations include small retrospective cohort, tissue-only analysis, lack of serum correlation, and absence of functional validation.	[159]
Pereira S.S. et al.	39 well-differentiated GEP-NET patients	Well-differentiated GEP-NET cohort including pancreatic NETs (*n* = 10) and gastrointestinal NETs (*n* = 29)	Well-differentiated NET G1/G2 only; panNETs: G1 *n* = 6, G2 *n* = 4; GI-NETs: G1 *n* = 24, G2 *n* = 5; no NET G3 or NECs were included	Formalin-fixed paraffin-embedded tumour and peritumoural tissue	Immunohistochemistry for IL-6, Ki-67, FOXM1, and IGF1R; IL-6 quantified in peritumoural tissue within ≤5 mm and >5 mm from the tumour margin using computerized morphometric analysis	Association of peritumoural IL-6 expression with metabolic syndrome features, tumour characteristics, and disease progression	In well-differentiated GEP-NETs, peritumoural IL-6 expression was not significantly influenced by metabolic syndrome overall. In GI-NETs, higher peritumoural IL-6 expression was associated with low HDL cholesterol and with disease progression, whereas no comparable association was shown for panNETs.	Findings suggest that local peritumoural IL-6 expression may be related to GI-NET behaviour and progression, but conclusions should be restricted to well-differentiated G1/G2 GEP-NETs, particularly GI-NETs. Results should not be extrapolated to NET G3 or poorly differentiated NECs; limitations include small single-centre cohort, tissue-only/peritumoural assessment, lack of serum cytokine correlation, and limited subgroup size.	[178]
Karakaxas D. et al.	51 pNET patients; comparator groups: 85 PDAC patients, 19 IPMN patients, and 98 healthy controls.	Sporadic pancreatic NETs, including functional pNETs (*n* = 29) and non-functional pNETs (*n* = 22); tumour location: pancreatic head *n* = 19, body/tail *n* = 32	pNET cohort with G1 *n* = 35, G2 *n* = 14, and G3 *n* = 2; NET versus NEC differentiation status of G3 cases was not explicitly separated	Germline DNA from peripheral blood	PCR-RFLP genotyping of IL1B −511 C/T (rs16944) and +3954 C/T (rs1143634) polymorphisms	Genetic susceptibility to sporadic pNET development; comparison with PDAC, IPMN, and healthy controls	IL1B −511 CT and TT high-expression genotypes, as well as the T allele, were overrepresented in pNET patients and associated with increased pNET risk. No significant association was found between IL1B genotypes or haplotypes and clinicopathological characteristics.	Findings support a possible association between IL1B −511 genetic variation and susceptibility to sporadic pNETs, but do not establish IL-1β as a circulating or tissue biomarker, nor as a prognostic marker. Conclusions should be limited to pNET susceptibility and should not be extrapolated to GEP-NETs from other sites or to poorly differentiated NECs; limitations include small sample size, genetic-only design, and lack of cytokine expression or serum validation.	[179]
Cigrovski Berković M. et al.	60 pNET patients; 60 healthy controls	Pancreatic NET cohort including functional pNETs (*n* = 23) and non-functional pNETs (*n* = 37)	pNETs described as mostly well-differentiated neuroendocrine neoplasms; tumour grade and NET versus NEC differentiation status were not reported in the cohort characteristics	Germline DNA from peripheral blood; serum	Real-time PCR-SNP analysis of IL1B −511 C/T (rs16944) and +3954 C/T (rs1143634) polymorphisms; serum IL-1β measured by chemiluminescent immunometric assay	Genetic susceptibility to functional and non-functional pNET development; exploratory serum IL-1β assessment	IL1B −511 C/T was associated with susceptibility to pNETs, particularly functional pNETs. The −511/+3954 CTCT genotype combination was associated with functional pNET risk, whereas CTCC was associated with non-functional pNET risk. Serum IL-1β levels were below the detection limit in all patients.	Findings support a possible association between IL1B polymorphisms and pNET susceptibility, but they do not establish circulating IL-1β as a diagnostic or prognostic biomarker. Conclusions should be limited to pNET susceptibility and should not be extrapolated to other GEP-NET sites, NET G3, or poorly differentiated NECs; limitations include small single-population cohort, lack of tumour tissue IL-1β validation, and absence of survival or progression endpoints.	[164]
Cigrovski Berković M. et al.	80 GEP-NET patients; 162 healthy controls	GEP-NET cohort including pancreatic endocrine tumours/panNETs (*n* = 39: functioning *n* = 17, non-functioning *n* = 22) and gastrointestinal NETs (*n* = 41)	Tumours were described as GEP-NETs/PETs; WHO grade and NET versus NEC differentiation status were not reported	Germline DNA from peripheral blood; serum	PCR-NlaIII RFLP analysis of IL6 −174 C/G promoter polymorphism; serum IL-6 measured by enzymatic solid-phase chemiluminescent immunometric assay	Association of IL6 −174 genotypes with GEP-NET subtype and serum IL-6 levels; comparison between functioning and non-functioning pancreatic NETs and gastrointestinal NETs	Overall IL6 −174 genotype distribution did not differ significantly between all GEP-NET patients and healthy controls. High-expression IL6 −174 CG/GG genotypes were more frequent in non-functioning pancreatic NETs than in functioning pancreatic NETs. Elevated serum IL-6 was observed in 36.8% of GEP-NET patients, predominantly in non-functioning pancreatic NETs, and serum IL-6 levels correlated with IL6 −174 genotype in non-functioning pancreatic NETs and gastrointestinal NETs.	Findings suggest a possible association between IL6 −174 genetic variation, serum IL-6 levels, and non-functioning pancreatic NET biology, but do not establish IL-6 as a validated prognostic marker. Conclusions should be limited to GEP-NETs, particularly non-functioning pancreatic NETs, and should not be extrapolated to NET G3 or poorly differentiated NECs; limitations include small subgroup sizes, lack of WHO grade reporting, absence of tumour tissue IL-6 validation, and no survival/progression endpoint.	[165]

Abbreviations: 5-HIAA, 5-hydroxyindoleacetic acid; BP-NETs, bronchopulmonary neuroendocrine tumours; CgA, chromogranin A; CT1, cardiotrophin-1; DNA, deoxyribonucleic acid; ELISA, enzyme-linked immunosorbent assay; FOXM1, forkhead box M1; G1, grade 1; G2, grade 2; G3, grade 3; GEP-NENs, gastroenteropancreatic neuroendocrine neoplasms; GEP-NETs, gastroenteropancreatic neuroendocrine tumours; GI-NETs, gastrointestinal neuroendocrine tumours; HDL, high-density lipoprotein; IGF1R, insulin-like growth factor 1 receptor; IL-1β, interleukin-1 beta; IL-6, interleukin-6; IL1B, interleukin-1 beta gene; IL6, interleukin-6 gene; IPMN, intraductal papillary mucinous neoplasm; Ki-67, proliferation marker Ki-67; NECs, neuroendocrine carcinomas; NENs, neuroendocrine neoplasms; NETest, neuroendocrine tumour transcriptomic blood test; NETs, neuroendocrine tumours; OSM, oncostatin M; PCR, polymerase chain reaction; PCR-RFLP, polymerase chain reaction–restriction fragment length polymorphism; PDAC, pancreatic ductal adenocarcinoma; PETs, pancreatic endocrine tumours; pNETs, pancreatic neuroendocrine tumours; RFLP, restriction fragment length polymorphism; SNP, single-nucleotide polymorphism; TNF-α, tumour necrosis factor-alpha; TNM, tumour-node-metastasis.

## Data Availability

No new datasets were generated or analyzed during the preparation of this narrative review. All data discussed in this manuscript were obtained from previously published studies cited in the reference list.
